# Trajectories of depressive symptoms of mothers and fathers over 11 years

**DOI:** 10.1017/S2045796025000174

**Published:** 2025-04-10

**Authors:** Zsófia Csajbók, Jakub Fořt, Pavla Brennan Kearns

**Affiliations:** 1 Department of Psychology and Life Sciences, Faculty of Humanities, Charles University, Prague, Czech Republic; 2 Department of Zoology, Faculty of Science, Charles University, Prague, Czech Republic; 3 Department of Epidemiology, Second Faculty of Medicine, Charles University, Prague, Czech Republic

**Keywords:** depressive symptoms, dyadic trajectories, latent trajectories, postpartum depression, relationship maintenance

## Abstract

**Aims**. Parental postpartum depressive symptoms have been extensively studied, but the combined longitudinal depression trajectories of parents and their long-term development beyond the postpartum period remain largely underexplored. We identified dyadic longitudinal depressive symptom trajectories in new parents, followed over an 11-year period, and compared parental characteristics, as well as child temperament and mental health factors, across different parental trajectory classes.

**Methods**. A prenatal cohort of 5,518 couples was studied. Depressive symptoms were measured using the Edinburgh Postnatal Depression Scale at eight time points: in the prenatal stage, in the newborn stage, and at 6 months, 18 months, 3 years, 5 years, 7 years and 11 years after the birth of the child.

**Results**. Dyadic Latent Class Growth Modelling identified five classes of couples: (1) mother has elevated depressive symptoms, father is non-depressed (24%); (2) both mother and father have elevated depressive symptoms (20%); (3) both mother and father are constantly non-depressed (42%); (4) both mother and father are constantly depressed (5%); and (5) mother is constantly depressed, father has elevated depressive symptoms (9%). Relationship maintenance (particularly being married or separated) was the most strongly associated with the classes. Socio-economic resources, emotional well-being, health, obstetric history and parental background also served as meaningful covariates. Child temperament and mental health showed weak correlations with parental trajectory classes.

**Conclusions**. Parents with postpartum depressive symptoms often experience depressive symptoms long-term. Separated parents are particularly vulnerable to adverse depressive trajectories. Our findings underscore the importance of dyadic methods in estimating unique combinations of parental depression trajectories.

## Introduction

Postpartum depression (PPD) is a serious mental health issue, affecting about 17% of mothers (Shorey *et al.*, 2018) and 9% of fathers (Rao *et al.*, 2020). PPD is a major depressive episode occurring during pregnancy or up to 4 months postpartum (American Psychiatric Association, 2013), but a universally accepted definition of PPD is lacking (Abbasi *et al.*, 2024). Individual studies relate it freely to a period they consider relevant for assessing PPD symptoms (e.g., Baron *et al.*, 2017).

PPD risk factors in both mothers and fathers include low social support (Dadi *et al.*, 2020; McCall-Hosenfeld *et al.*, 2016), history of depression (McCall-Hosenfeld *et al.*, 2016; Tebeka *et al.*, 2016) and low marital satisfaction (Escriba-Aguir and Artazcoz, 2011; Wang *et al.*, 2021). Especially for fathers, these risk factors also include unemployment and financial strain (Serhan *et al.*, 2013; Wang *et al.*, 2021), while low economic and educational status were also identified for mothers (Dadi *et al.*, 2020; McCall-Hosenfeld *et al.*, 2016). Adverse obstetric history correlated with PPD in mothers (Dadi *et al.*, 2020), and newborn’s impaired health correlated with PPD in both mothers and fathers (Genova *et al.*, 2022).

PPD may have important adverse outcomes in children including malnutrition, common infant illness, poorer parent–child bonding and non-exclusive breastfeeding (Dadi *et al.*, 2020; Handelzalts *et al.*, 2024). Children in families where both parents or at least mothers experience PPD show higher amount of externalizing and internalizing problems under 5 years of age (Pietikäinen *et al.*, 2020; Volling *et al.*, 2019). However, results are mixed about whether PPD of parents influences the motor and language skills or cognitive development of their children (Aoyagi *et al.*, 2019; Aoyagi and Tsuchiya, 2019).

Previous research on PPD risk factors often utilized cross-sectional design on the overall sample (e.g., Wang *et al.*, 2021; Yang *et al.*, 2022). In contrast, the state-of-the-art approach relies on identifying patterns in the data where subgroups of individuals or couples follow unique (e.g., progressing or regressing) longitudinal depressive symptom trajectories (e.g., Csajbók *et al.*, 2022; Formánek *et al.*, 2020). In a systematic review, the commonest classes found in mothers were (1) a large, stable, low-risk trajectory with minimal symptoms; (2) a small, stable, high-risk trajectory with severe symptomatology; and (3) a stable or transient trajectory with variously serious symptoms (Baron *et al.*, 2017). Prior psychiatric diagnosis, singlehood, post birth complications, alcohol and tobacco use and hypertension increased the likelihood of belonging to stable high trajectories, while having social support decreased it (Drozd *et al.*, 2018; Handelzalts *et al.*, 2024; Hong *et al.*, 2022). Few studies examined possible PPD trajectories in fathers, but they found similar patterns: low, moderate and high PPD, with financial status being a risk factor (Molgora *et al.*, 2017; Nieh *et al.*, 2022). Shared risk factors between mothers and fathers were insomnia, anxiousness, earlier depression, stressfulness and poor family atmosphere (Kiviruusu et al., 2020). One study identified four joint, dyadic trajectories: both mother and father non-depressed; both mother and father depressed; mother non-depressed, father depressed; and mother depressed, father non-depressed (Volling *et al.*, 2019). When both parents were depressed, they had worse marital quality, and their child demonstrated more externalizing and internalizing problems.

We compared how overall PPD symptoms and depressive symptom trajectories correlate with PPD risk factors and offspring outcomes in a large, well-characterized prenatal cohort of new parents in Czechia. Since PPD can be a precursor of long-term depressive symptoms in parents (Bloch *et al.*, 2024), we uniquely followed parents from the prenatal period until their child was 11 years old. Unlike much of the previous research, we used a dyadic, parallel processes approach to study couples, allowing us to estimate unique combinations of maternal and paternal trajectories in a data-driven manner. By exploring the heterogeneity among couples, we aimed to better identify risk factors that are uniquely applicable to different family trajectory classes.

## Methods

### Participants and procedure

We analysed data from the Czech arm of the international European Longitudinal Study of Pregnancy and Childhood (ELSPAC-CZ; Piler *et al.*, 2016). ELSPAC-CZ is a prenatal cohort following children born between 1991 and 1992 in two towns in Czechia (Brno, Znojmo). Mothers were enrolled in the second or the third trimester. Mothers and fathers filled in several questionnaires about themselves and their child, including repeated assessments of their mental health. Presently, we use data from questionnaires administered to the parents at the following time points: in the prenatal period, in the newborn stage and at 6 months, 18 months, 3 years, 5 years, 7 years and 11 years of age of the child. Additionally, we use information acquired from the children themselves at the ages of 11, 15, 18 and 19 years. We excluded mothers and fathers who did not have any measures of depressive symptoms and those who were not biological parents of the children, leaving a sample of 5,518 families. All participants provided written informed consent. Ethical approval was obtained from the ELSPAC-CZ Ethics Committee.

## Measures

### Depressive symptoms

Depressive symptoms were assessed using the Edinburgh Perinatal/Postnatal Depression Scale (EPDS; Cox *et al.*, 1987), which was administered to the mothers and fathers at all follow-up points. The measure has been validated for non-postnatal populations (Becht *et al.*, 2001; Cox *et al.*, 1996). The scale consists of 10 items concerning the experience of symptoms of depression during the past week. The parents reported to what extent they have experienced the following: ability to laugh, looking forward to things, feeling guilty, being worried, feeling panicky, feeling that things have been getting on top of them, difficulty sleeping, feeling sad, crying, thought of harming oneself. Symptoms were assessed on a 0–3 scale. The final score is the sum of the points from the individual items, where higher values indicate greater severity of depressive symptoms. We considered the cut-off for *PPD* to be 11 and above (Levis *et al.*, 2020). We also relied on a general risk assessment for *elevated depressive symptoms* using 5 as a cut-off (Matthey *et al.*, 2001).

### Covariates

We selected psychosocial, health-related and obstetrical covariates available in the ELSPAC database based on existing literature, focusing on characteristics of parents and offspring that have been linked to parental depressive symptoms (Guintivano *et al.*, 2018) or offspring psychological outcomes (Pietikäinen *et al.*, 2020). We grouped the selected covariates into eight areas as follows: *relationship maintenance, demographic data, socio-economic resources, emotional life, health, obstetric history, parental history*, and *offspring temperament and mental health*. The data on all areas except offspring mental health were reported by the mothers and the fathers in several questionnaires throughout the first 11 years. Here, we consider the information from baseline; if this information was not available in the questionnaire from the prenatal stage, it was taken from the next one, when the information was first available.

*Relationship maintenance* was categorized in multiple ways: unstable relationship, only married, continuously married, married ever, married later, other than first marriage (remarriage), bereavement, separated, divorced, single (at any point during the study) and continuously cohabitated (no indication of living apart). *Demographic data* included age of the parents, child’s sex, town (Brno, Znojmo) and education. Data on *socio-economic resources* concerned income, crowding, deprivation, father’s employment, financial help and living in own house. We also included information about social network and social support. Further information was reported by the mother of the child at 6 months on childcare provided by other people (father, other family members, someone who is not family) expressed in hours per week and the age of the child (in months) when they started to take care of the child.

Information about *emotional life* concerned relationship aggression, affection and love of the baby (how long it took the mother to love the baby). Information about *health* concerned the number of diseases, substance use, smoking, alcohol use and use of psychotropic pills. *Obstetric history* included data about previous pregnancy, number of previous pregnancies, number of own children, history of miscarriage, number of miscarriages, history of abortion, number of abortions and obstetric treatment for getting pregnant.

Information about *parental history* includes stressful life events (sum of 41 events since pregnancy), parental care (derived from the Parental Bonding Instrument, PBI; Parker *et al.*, 1979), overprotection (derived from the PBI items), home stability and sexual abuse. *Offspring temperament* was assessed in the newborn questionnaire, in which parents rated 14 items based on how much the child shows the characteristics of being whiny, cranky, satisfied, etc. *Offspring mental health* was completed by the child and included internalizing and externalizing symptoms (derived from the Strengths and Difficulties Questionnaire; Goodman, 1997), satisfaction with life (Diener *et al.*, 1985) and stressful life events (year 11: 20 events, year 15: 28 events, year 18 and 19: 33 events). Cronbach’s alphas of all scales ranged between 0.65 and 0.90, which is considered an acceptable level of internal consistency (Tables S1 and S6; Bland and Altman, 1997). The detailed definitions of each covariate with rating scales are presented in Supplementary Measures.

### Data analysis

Descriptive characteristics are presented as mean and standard deviation or frequency (*n*, %). Parental longitudinal dyadic depression trajectories were extracted with Latent Class Growth Modelling method (see Supplementary Data analysis). This is a probabilistic method that can identify subgroups in our sample, based on which couples are most likely to follow similar longitudinal trajectories. The Latent Base Growth Model (LBGM) that allows for any shape of trajectories fit better the data than the simple linear growth model (and the curved growth model did not converge), thus we submitted the LBGM to the mixture modelling for classification (Table S2). Relying on the combination of model indices and interpretability, the five-class model was selected for further analysis (Tables S3–S5).

In Methods, we listed all covariates tested, and in Results, only class comparisons across covariates with meaningful associations were mentioned. In the Supplement, (1) the overall sample was correlated with all covariates and we reported only those, which had meaningful associations (Supplementary Results, Tables S7 and S8), and (2) results of class comparisons with weak associations are presented in supplementary tables (Tables S10–S12). The covariates selected for reporting in the main text were identified based on effect size (*η*^2^ > .02, or *V* > .04). The selected parental and offspring characteristics, if they were continuous variables, were compared between the five classes of couples with Brown–Forsythe ANOVA and Games–Howell *post hoc* tests due to unequal variances and class sizes. *χ*^2^ tests were used to compare categorical parental characteristics between classes.

## Results

**Figure 1.**
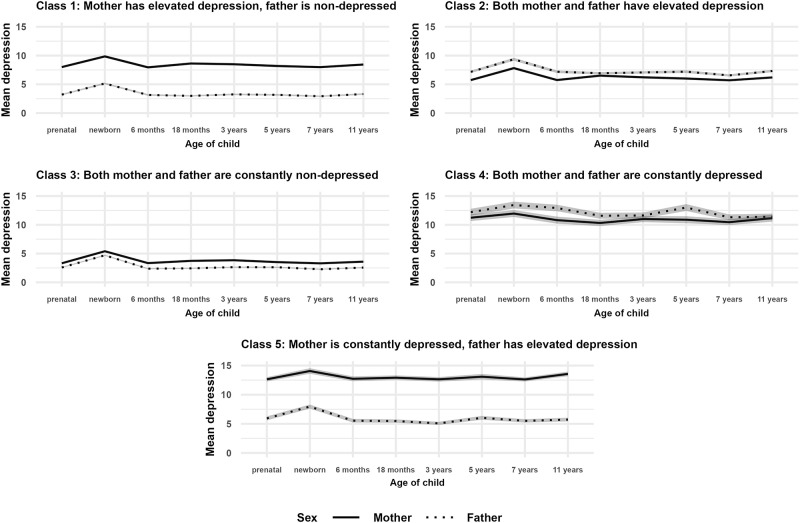
Five classes of parental dyadic depression trajectories presenting mean depressive symptom scores at each time point across mothers and fathers with 95% confidence intervals (shadowed with grey).

Descriptive statistics and data coverage of the depressive symptoms scores are presented in Table S1. In the overall sample, baseline parental characteristics, along with offspring temperament and mental health, were examined for associations with parental depressive symptoms at each time point using independent samples *t*-tests and Pearson correlations (see Supplementary Results and Supplementary Discussion). The five-class model of dyadic longitudinal depressive symptom trajectories showed the following patterns of dyads: Class 1 mother has elevated depressive symptoms, father is non-depressed (24.19%); Class 2 both mother and father have elevated depressive symptoms (19.61%); Class 3 both mother and father are constantly non-depressed (42.30%); Class 4 both mother and father are constantly depressed (4.93%); and Class 5 mother is constantly depressed, father has elevated depressive symptoms (8.97%), see Table 1 and Figure 1.

**Table 1.** Proportions of couples and model results of the five-class solution

	*N* of class members	% of total *N*	Mean maternal latent intercept factor	Mean maternal latent slope factor	Mean paternal latent intercept factor	Mean paternal latent slope factor
Class 1	1335	24.19	8.24***	1.54***	3.20***	2.02***
Class 2	1082	19.61	6.23***	1.68***	6.89***	2.48***
Class 3	2334	42.30	3.79***	1.65***	2.54***	2.10***
Class 4	272	4.93	10.82***	0.83	11.78***	1.85*
Class 5	495	8.97	12.82***	1.07*	5.54***	2.20***

*Note*: Variables in the analyses were divided by three to aid model convergence (following the common practice using Mplus software). We converted these results back to the original scaling (i.e., we reported here three times the results presented in Table S4) to help interpretation using the more familiar scoring of the Edinburgh Postnatal Depression Scale.

* *p* < .05, ***p < .001.

Descriptive statistics and data coverage of the covariates are in Table S6. When we compared the five classes of parents along the selected covariates, the classes differed in relationship stability with the non-depressed Class 3 being the least likely unstable and the depressed Classes 4 and 5 being the most likely unstable, while Classes 1 and 2 were in-between (*V* = .11, Table 2). Most notably, Class 3 was the most likely married (only married *V* = .13 and continuously married *V* = .11) and the least likely separated (*V* = .14). Class 4 was the least likely married and the most likely divorced considering the entire study period (*V* = .09). Related to socio-economic resources, we found notable differences in deprivation. Based on the *post hoc* test, the non-depressed Class 3 was the least deprived and the depressed Classes 4 and 5 were the most deprived, while Classes 1 and 2 were in-between (*η*^2^ = .04). Fathers were the most likely working if non-depressed (i.e., Classes 1 and 3). Both mothers (*η*^2^ = .06) and fathers (*η*^2^ = .06) enjoyed the most amount of social support in the non-depressed Class 3 and the least amount of support in the depressed Classes 4 and 5, while Classes 1 and 2 were found in-between.

**Table 2.** Brown–Forsythe analysis of variance tests and *χ*^2^ tests comparing the five classes along selected parental characteristics

	Class 1: Mother has elevated depression, father is non-depressed	Class 2: Both mother and father have elevated depression	Class 3: Both mother and father are constantly non-depressed	Class 4: Both mother and father are constantly depressed	Class 5: Mother is constantly depressed, father has elevated depression	*η*^2^ or *V*
Relationship maintenance						
Unstable relationship *n*, %	184 (13.8)	163 (15.1)	264 (11.3)	68 (25.2)	111 (22.4)	.11***
Continuously married *n*, %	752 (56.3)	560 (51.8)	1276 (54.7)	93 (34.2)	210 (42.4)	.11***
Only married *n*, %	911 (68.2)	693 (64)	1595 (68.3)	128 (47.1)	258 (52.1)	.13***
Married ever *n*, %	1209 (92.6)	960 (91.7)	2094 (92.9)	213 (84.2)	418 (86.5)	.09***
Not first marriage *n*, %	182 (13.9)	167 (16)	354 (15.7)	59 (23.3)	99 (20.5)	.06***
Bereavement *n*, %	29 (2.2)	19 (1.8)	23 (1.0)	7 (2.7)	10 (2.1)	.04*
Separated *n*, %	56 (4.3)	61 (5.8)	58 (2.6)	29 (11.5)	57 (11.8)	.14***
Divorced *n*, %	116 (8.9)	108 (10.3)	178 (7.9)	43 (17)	77 (15.9)	.09***
Single ever *n*, %	118 (9)	98 (9.4)	204 (9.1)	38 (15)	57 (11.8)	.05*
Continuous cohabitation *n*, %	1193 (91.3)	942 (90.4)	2075 (92)	201 (79.1)	410 (85.4)	.10***
Socio-economic resources						
Deprivation *M, SD*	8.24 (3.15)	8.12 (3.06)	7.48 (3.02)	8.99 (3.71)	9.52 (3.7)	.04***
Employed (f.) *n*, %	1038 (93.9)	791 (89.6)	1738 (93.1)	175 (86.2)	359 (91.8%)	.08***
Social network (m.) *M, SD*	21.08 (4.13)	21.16 (4.2)	21.75 (3.91)	19.72 (4.33)	20.12 (3.98)	.02***
Social network (f.) *M, SD*	21.70 (4.01)	20.76 (4.57)	21.95 (4.03)	19.24 (4.87)	20.65 (4.29)	.03***
Social support (m.) *M, SD*	16.99 (4.03)	17.09 (3.92)	18.36 (3.82)	15.28 (4.42)	15.62 (4.33)	.06***
Social support (f.) *M, SD*	19.67 (4.29)	17.92 (4.68)	20.24 (4.22)	16.39 (4.48)	17.99 (4.34)	.06***

*Note*: m = mother, f = father, *M* = mean, *SD* = standard deviation.

* *p* < .05, ***p < .001.

Aggression (mother *η*^2^ = .06, father *η*^2^ = .05) was the least pronounced in Class 3 and the most noticeable in Classes 4 and 5, while affection (mother *η*^2^ = .05, father *η*^2^ = .04) was the strongest in Class 3 and the weakest in Classes 4 and 5 (Table 3). Aggression and affection levels in Classes 1 and 2 were consistently found between Class 3 and Classes 4 and 5. We also found differences in the number of diseases in fathers (*η*^2^ = .03) with the non-depressed Class 3 experiencing the least and the depressed Classes 4 and 5 the greatest number of diseases, while Classes 1 and 2 were found in-between. Similarly, mothers used psychotropic pills the least amount in Class 3 and the most in Classes 4 and 5, while Classes 1 and 2 were in-between (*η*^2^ = .03). Substance use in Class 4 fathers was the most prevalent and in Class 2 the second most prevalent (*V* = .11). Class 3 parents experienced the least amount of stressful life events and Classes 4 and 5 parents were influenced the most by such events, with Classes 1 and 2 being in-between (mother *η*^2^ = .09, father *η*^2^ = .07). We also found that the non-depressed Class 3 parents evaluated their baby the least cranky (*η*^2^ = .03) and the depressed Classes 4 and 5 evaluated their baby the crankiest, with Classes 1 and 2 in-between. The children of Class 3 were the least influenced by stressful life events at the age of 11 (*η*^2^ = .03) and 19 years (*η*^2^ = .04), while children in Class 5 experienced the most stressful life events at age 11 and Class 4 at age 19.

**Table 3.** Brown–Forsythe analysis of variance tests and *χ*^2^ tests comparing the five classes along selected parental and offspring characteristics

	Class 1: Mother has elevated depression, father is non-depressed	Class 2: Both mother and father have elevated depression	Class 3: Both mother and father are constantly non-depressed	Class 4: Both mother and father are constantly depressed	Class 5: Mother is constantly depressed, father has elevated depression	*η*^2^ or *V*
Emotional life						
Aggression (m.) *M, SD*	7.61 (1.77)	7.61 (1.75)	6.91 (1.75)	8.38 (2.06)	8.19 (1.85)	.06***
Aggression (f.) *M, SD*	7.60 (1.9)	7.89 (1.82)	6.95 (1.83)	8.24 (2.04)	7.94 (1.99)	.05***
Affection (m.) *M, SD*	23.45 (3.68)	23.44 (3.62)	24.70 (3.4)	22.25 (3.93)	22.48 (3.87)	.05***
Affection (f.) *M, SD*	23.88 (3.62)	23.26 (3.64)	24.81 (3.45)	22.95 (3.78)	23.35 (3.79)	.04***
Health						
*N* of diseases (m.) *M, SD*	0.79 (1.08)	0.71 (1.08)	0.6 (0.92)	0.78 (1.15)	1.03 (1.27)	.01***
*N* of diseases (f.) *M, SD*	0.62 (0.92)	0.86 (1.11)	0.52 (0.84)	1.16 (1.52)	0.92 (1.58)	.03***
Substance use (m.) *n*, %	17 (1.6)	13 (1.6)	26 (1.4)	9 (4.9)	15 (4.2)	.07***
Substance use (f.) *n*, %	30 (2.3)	52 (5.0)	42 (1.9)	25 (10)	17 (3.7)	.11***
Smoking (m.) *n*, %	180 (15.4)	164 (17.4)	295 (14.3)	61 (26.9)	102 (23.9)	.09***
Smoking (f.) *n*, %	441 (43)	365 (46.1)	781 (42.8)	93 (53.8)	176 (50.1)	.06**
Psychotropic pills (m.) *M, SD*	1.06 (0.29)	1.04 (0.21)	1.02 (0.16)	1.13 (0.41)	1.19 (0.52)	.03***
Psychotropic pills (f.) *M, SD*	1.03 (0.2)	1.07 (0.33)	1.01 (0.12)	1.13 (0.46)	1.04 (0.21)	.02***
Obstetric history						
Previous pregnancy *n*, %	664 (61.6)	554 (65.9)	1120 (59.2)	131 (68.2)	244 (66.1)	.06**
Miscarriage *n*, %	138 (20.8)	147 (26.6)	289 (25.9)	32 (24.4)	56 (23)	.05
Abortion *n*, %	232 (35)	200 (36.2)	372 (33.3)	59 (45)	112 (46.3)	.09***
Obs. treatment *n*, %	32 (30.2)	24 (30.8)	67 (40.9)	5 (23.8)	14 (32.6)	.11
Parental history						
Stressful life events (m.) *M, SD*	3.28 (2.94)	3.14 (2.87)	2.23 (2.02)	4.56 (3.27)	4.79 (3.73)	.09***
Stressful life events (f.) *M, SD*	3.03 (2.48)	3.78 (3.27)	2.40 (2.28)	5.36 (4.42)	3.96 (2.81)	.07***
Offspring temp. & mental health						
Cranky baby *M, SD*	1.65 (0.68)	1.59 (0.67)	1.45 (0.59)	1.72 (0.73)	1.73 (0.72)	.03***
Stressful life events (11y) *M, SD*	0.47 (0.37)	0.45 (0.35)	0.39 (0.33)	0.47 (0.39)	0.59 (0.42)	.03***
Stressful life events (19y) *M, SD*	0.34 (0.28)	0.36 (0.36)	0.30 (0.25)	0.57 (0.38)	0.41 (0.29)	.04**

*Note*: m = mother, f = father, *M* = mean, *SD* = standard deviation, Obs. treatment = obstetric treatment to get pregnant. Temp = temperament.

** *p* < .01, ***p < .001.

Among relationship maintenance variables only being married later was not a meaningful covariate (Table S10). Demographic data like age, education, town, child’s sex; socio-economic resources like living in own house, needing financial help, income, crowding, the amount of childcare provided by the father, other family and non-family did not differ substantially across the classes. Love of the baby (emotional life covariate) did not correlate with the trajectories either. Obstetric history, such as the number of previous pregnancies, miscarriages or abortions did not differ substantially. Similarly, alcohol use (health covariate) had a negligible association. Temperamental and mental health characteristics of the offspring (apart from a cranky baby), such as internalizing and externalizing problems, self-esteem and satisfaction with life were only negligibly associated with parental depressive symptom trajectories (Tables S11 and S12).

## Discussion

We identified five distinct classes of parental depression trajectories. First, mother has elevated depressive symptoms, father is non-depressed; second, both mother and father have elevated depressive symptoms; third, both mother and father are non-depressed; fourth, both mother and father are depressed; and fifth, mother is constantly depressed, father has elevated depressive symptoms. Importantly, the levels of depressive symptoms were maintained during the entire study period from before the birth of the child till the child’s age of 11 years. Further, understandably, we can see a spike in depressive symptoms at newborn age in essentially all the classes. Notably, while the mean latent slope factors were all positive and non-zero, we did not interpret the pace of change due to inconsistent measurement intervals (i.e., prenatal, newborn, 6 months, 18 months, etc.), making it difficult to assess the rate of increase accurately. Comparing the five classes of parents, we found that only some studied characteristics, such as relationship maintenance, some indicators of socio-economic resources, emotional life, health, obstetric history and having a cranky baby were associated with increased levels of depressive symptoms after childbirth in both mothers and fathers. Offspring of more depressed parents were more likely to experience more stressful life events at the ages of 11 and 19 years. Additionally, similar variables were found to be important covariates in the overall sample as across the classes.

Surprisingly, several established socio-demographic and socio-economic risk factors for depression – such as age, education and wealth indicators (home ownership, financial need, income, crowding) – were not associated with parental depressive symptom trajectories in our study, contrasting with prior research (Cermakova and Csajbók, 2023; McCall-Hosenfeld *et al.*, 2016; Yang *et al.*, 2022). In our study, only deprivation – likely reflecting the experience of poverty – was linked to parental depressive trajectories, suggesting that other wealth-related markers are not meaningful predictors of parents’ depressive symptom development over time. Conversely, we found that the strength of parents’ social network and the support they receive are significant predictors of future depressive symptom trajectories, supporting the view that social support plays a crucial role in mitigating the stressors faced by parents (Escriba-Aguir and Artazcoz, 2011; McCall-Hosenfeld *et al.*, 2016). Contrary to the notion that childcare responsibilities predominantly falling on mothers predict future mental health problems (Serhan et al., 2013), our findings do not suggest that childcare provided by others significantly impacts long-term parental depressive trajectories in Czech parents. Perhaps we found this because our measure only indicated the timing and duration of childcare provided instead of the quality and satisfaction with the help received or the associated childcare stress experienced (Ando *et al.*, 2021; Dennis *et al.*, 2018). Also, since social support and network was a significant alleviating factor, but not directly childcare, it is likely that Czech parents benefit from social support in some aspects other than childcare provision, such as time availability for personal care, emotional support and having adult conversations, exchanging information, or cooking and household maintenance (Chojenta *et al.*, 2012; Corrigan *et al.*, 2015).

Consistent with previous research (Beach *et al.*, 2003; Braithwaite and Holt-Lunstad, 2017), we found that the emotional quality of the relationship between parents – characterized by levels of aggression, affection and various indicators of relationship stability – strongly predicts the development of depressive symptoms. This highlights that intervention techniques such as family therapy improving relationship functioning between mothers and fathers should be a key focus of practical and clinical professionals (Cluxton-Keller and Bruce, 2018). Notably, the strongest association was found with being separated, emphasizing the significant emotional burden parents face during such challenging life transitions. It is likely that by the time parents divorce or reconcile, many of the difficulties have already been processed during the separation period, which may explain why having experienced a separation is associated with worse outcomes.

Previous studies on couples’ dyadic longitudinal depressive trajectories have either not identified couples where both partners are depressed (Csajbók *et al.*, 2022) or have done so (Volling *et al.*, 2019). In our study, we identified a small group where both mothers and fathers were constantly depressed. Notably, fathers in this group had the highest intercept score, nearly 12, among all classes. This highlights that while women face an elevated risk for depression throughout life (Lim *et al.*, 2018), men can also be vulnerable during sensitive periods, such as when caring for young children.

Previous research found a group of couples where women were constantly depressed and men were constantly non-depressed (Csajbók *et al.*, 2022; Pietikäinen *et al.*, 2020; Volling *et al.*, 2019). In our study, we also found couples who were different from each other, but not to such a great extent. In Class 1, women had elevated depressive symptoms and men were non-depressed. In Class 5, women had high depression, and men had elevated depressive symptoms. This is interesting, because couples generally tend to be similar in various traits, including depressiveness (Luo, 2017; Nordsletten *et al.*, 2016). Surprisingly, while Class 1 formed a stable relationship, Class 5 had an increased risk for dissolution. Class 5 was also deprived, had relatively little support, had worse emotional life and more stressful life events. Thus, the difference between the overall picture of Classes 1 and 5, alarmingly, is more than just 2–4 depression points. Notably, mothers in this group had the highest intercept score, almost 13 points, among all classes.

Similarly to previous studies (Csajbók *et al.*, 2022; Kiviruusu et al., 2020; Volling *et al.*, 2019), we also found that most couples are non-depressed, though this proportion is significantly lower postpartum compared to later life. Specifically, 41–42% of parents in both Volling *et al.* (2019) and our study reported no elevated depressive symptoms. In Kiviruusu et al. (2020), 63% of mothers and 75% of fathers were constantly non-depressed, while approximately 80% of older couples in Csajbók *et al.* (2022) fell into this category. This figure is alarming and highlights the vulnerability of families with small children in Czechia. Additionally, other studies identified groups of mothers and/or couples with either decreasing or increasing depressive symptoms (Baron *et al.*, 2017; Csajbók *et al.*, 2022). We did not find such a dynamic change in depressive symptoms except for the spike around the birth of the child. The lack of alleviating effects is worrisome and indicates that these couples are dealing with pervasive and persistent problems, as our data indicated no noticeable improvement over at least 11 years. Future research should explore why depressive symptoms remain stable (though not worsening) in Czech families with young children.

Findings are in accordance with the social risk hypothesis that posits that depressed mood is part of an adaptive strategy that protects the individual from acting dangerously and losing more social capital than one can afford (Allen and Badcock, 2003). Caring for young and dependent offspring requires significant psychological and physical investment from both mothers and fathers (Trivers, 1972). This investment is especially critical for parents in particularly vulnerable positions, such as the parents of Classes 4 and 5. It follows that these more depressed parents have fewer resources and privileges to draw upon, leading them to respond with depressive symptoms as an evolutionarily developed strategy. The associated covariates and outcomes of depressive trajectories, such as relationship and emotional problems, socio-economic deprivation, substance abuse and offspring mental health likely have a complex interplay among them (e.g., Amendola *et al.*, 2022; Boardman *et al.*, 2001; Ettekal *et al.*, 2020; Whisman, 2007). While it is more likely that marital distress is causing depressive symptoms, living with a depressed partner is also negatively affecting marital quality (Coyne, 1976; Gariépy *et al.*, 2016; Whisman and Bruce, 1999). Thus, while depressed mood serves important functions in a person’s life, professionals need to apply a nuanced and complex approach when helping families in need.

### Strengths and limitations

The strength of this study was that all families with a child born in Brno and Znojmo between 1991 and 1992 were invited to participate, and these families were very closely monitored essentially throughout the child’s life. Further, instead of simply correlating depression measures with various covariates, we could identify mothers and fathers who specifically experienced elevated or high depressive symptoms postpartum, focusing on estimating combined trajectories of the dyads. Based on this analysis, we showed that not only high depression, but elevated depressive symptoms could be a sign of relational and socio-economic hardship and an indicator of need for help. Several limitations need to be mentioned. There were missing data and a high attrition in the offspring data at ages 18 and above. Some of the covariates, such as smoking status, were also measured on an overly simplistic scale, thus limiting how much variance they could capture as a function of depressive symptoms. We had limited overlapping mental health data coming from both offspring and parents (only from the age of 11 years), which limits the predictive power of parental depression trajectories on children’s mental health outcomes. Future research should aim to gather longitudinal data that includes parallel assessments of all family members and to perform detailed psychometric analysis, including longitudinal measurement invariance testing of the depressive symptoms scale.

## Supporting information

Csajbók et al. supplementary material Csajbók et al. supplementary material

## Data Availability

Access to the data is provided free of charge to researchers upon reasonable request. More information can be found on this website: elspac.cz. The study protocol and syntax of the statistical analysis will be shared upon request from the corresponding author of this study.

## Supplementary material

The supplementary material for this article can be found at https://doi.org/10.1017/S2045796025000174.

## References

[ref1] Abbasi NUH, Bilal A, Muhammad K, Riaz S and Altaf S (2024) Relationship between personality traits and postpartum depression in Pakistani fathers. *PLoS ONE* 19, e0303474. 38743742 10.1371/journal.pone.0303474 PMC11093302

[ref2] Allen NB and Badcock PBT (2003) The social risk hypothesis of depressed mood: Evolutionary, psychosocial, and neurobiological perspectives. *Psychological Bulletin* 129, 887–913. 14599287 10.1037/0033-2909.129.6.887

[ref3] Amendola S, Hengartner MP, Ajdacic-Gross V, Angst J and Rössler W (2022) Longitudinal reciprocal associations between depression, anxiety, and substance use disorders over three decades of life. *Journal of Affective Disorders* 302, 315–323. 35093414 10.1016/j.jad.2022.01.101

[ref4] American Psychiatric Association (2013) Depressive disorders. In *Diagnostic and Statistical Manual of Mental Disorders*. 5th edition. Arlington, VA: American Psychiatric Publishing Inc., 153–188.

[ref5] Ando H, Shen J, Morishige KI, Suto S, Nakashima T, Furui T, Kawasaki Y, Watanabe H and Saijo T (2021) Association between postpartum depression and social support satisfaction levels at four months after childbirth. *Archives of Psychiatric Nursing* 35, 341–346. 34176574 10.1016/j.apnu.2021.03.010

[ref6] Aoyagi SS, Takei N, Nishimura T, Nomura Y and Tsuchiya KJ (2019) Association of late-onset postpartum depression of mothers with expressive language development during infancy and early childhood: The HBC study. *PeerJ* 7, e6566. 30863683 10.7717/peerj.6566 PMC6408909

[ref7] Aoyagi S and Tsuchiya KJ (2019) Does maternal postpartum depression affect children’s developmental outcomes? *Journal of Obstetrics & Gynaecology Research* 45, 1809–1820. 31321836 10.1111/jog.14064

[ref8] Baron E, Bass J, Murray SM, Schneider M and Lund C (2017) A systematic review of growth curve mixture modelling literature investigating trajectories of perinatal depressive symptoms and associated risk factors. *Journal of Affective Disorders* 223, 194–208. 28763638 10.1016/j.jad.2017.07.046 PMC5592733

[ref9] Beach SRH, Katz J, Kim S and Brody GH (2003) Prospective effects of marital satisfaction on depressive symptoms in established marriages: A dyadic model. *Journal of Social and Personal Relationships* 20, 355–371.

[ref10] Becht MC, Van Erp CF, Teeuwisse TM, Van Heck GL, Van Son MJ and Pop VJ (2001) Measuring depression in women around menopausal age: Towards a validation of the Edinburgh Depression Scale. *Journal of Affective Disorders* 63, 209–213. 11246097 10.1016/s0165-0327(99)00189-5

[ref11] Bland JM and Altman DG (1997) Statistics notes: Cronbach’s alpha. *BMJ (British Medical Journal)* 314, 572.9055718

[ref12] Bloch M, Tevet M, Onn R, Fried-Zaig I and Aisenberg-Romano G (2024) The long-term course and prognosis of postpartum depression: A retrospective longitudinal cohort study. *Archives of Women’s Mental Health* 27, 99–107. 10.1007/s00737-023-01373-6 37749279

[ref13] Boardman JD, Finch BK, Ellison CG, Williams DR and Jackson JS (2001) Neighborhood disadvantage, stress, and drug use among adults. *Journal of Health and Social Behavior* 42, 151–165. 11467250

[ref14] Braithwaite S and Holt-Lunstad J (2017) Romantic relationships and mental health. *Current Opinion in Psychology* 13, 120–125. 28813281 10.1016/j.copsyc.2016.04.001

[ref15] Cermakova P and Csajbók Z (2023) Household crowding in childhood and trajectories of depressive symptoms in mid-life and older age. *Journal of Affective Disorders* 340, 456–461. 37573892 10.1016/j.jad.2023.08.056

[ref16] Chojenta C, Loxton D and Lucke J (2012) How do previous mental health, social support, and stressful life events contribute to postnatal depression in a representative sample of Australian women? *Journal of Midwifery & Women’s Health* 57, 145–150. 10.1111/j.1542-2011.2011.00140.x 22432486

[ref17] Cluxton-Keller F and Bruce ML (2018) Clinical effectiveness of family therapeutic interventions in the prevention and treatment of perinatal depression: A systematic review and meta-analysis. *PL*O*S ONE* 13, e0198730. 29902211 10.1371/journal.pone.0198730 PMC6002098

[ref18] Corrigan CP, Kwasky AN and Groh CJ (2015) Social support, postpartum depression, and professional assistance: A survey of mothers in the Midwestern United States. *The Journal of Perinatal Education* 24, 48–60. 26937161 10.1891/1058-1243.24.1.48 PMC4720860

[ref19] Cox JL, Chapman G, Murray D and Jones P (1996) Validation of the Edinburgh postnatal depression scale (EPDS) in non-postnatal women. *Journal of Affective Disorders* 39, 185–189. 8856422 10.1016/0165-0327(96)00008-0

[ref20] Cox JL, Holden JM and Sagovsky R (1987) Detection of postnatal depression: Development of the 10-item Edinburgh Postnatal Depression Scale. *British Journal of Psychiatry* 150, 782–786. 10.1192/bjp.150.6.782 3651732

[ref21] Coyne JC (1976) Depression and the response of others. *Journal of Abnormal Psychology* 85, 186–193. 1254779 10.1037//0021-843x.85.2.186

[ref22] Csajbók Z, Štěrbová Z, Jonason PK, Cermakova P, Dóka Á and Havlíček J (2022) Variation in depressive symptom trajectories in a large sample of couples. *Translational Psychiatry* 12, 206–211. 35581177 10.1038/s41398-022-01950-w PMC9113986

[ref23] Dadi AF, Miller ER and Mwanri L (2020) Postnatal depression and its association with adverse infant health outcomes in low- and middle-income countries: a systematic review and meta-analysis. *BMC Pregnancy & Childbirth* 20, 416–430. 32698779 10.1186/s12884-020-03092-7 PMC7374875

[ref24] Dennis CL, Brown HK and Brennenstuhl S (2018) Development, Psychometric Assessment, and Predictive Validity of the Postpartum Childcare Stress Checklist. *Nursing Research* 67, 439–446. 30067584 10.1097/NNR.0000000000000308

[ref25] Diener E, Emmons RA, Larsen RJ and Griffin S (1985) The Satisfaction With Life Scale. *Journal of Personality Assessment* 49, 71–75. 16367493 10.1207/s15327752jpa4901_13

[ref26] Drozd F, Haga SM, Valla L and Slinning K (2018) Latent trajectory classes of postpartum depressive symptoms: A regional population-based longitudinal study. *Journal of Affective Disorders* 241, 29–36. 30096589 10.1016/j.jad.2018.07.081

[ref27] Escriba-Aguir V and Artazcoz L (2011) Gender differences in postpartum depression: A longitudinal cohort study. *Journal of Epidemiology & Community Health* 65, 320–326. 20515899 10.1136/jech.2008.085894 PMC3069755

[ref28] Ettekal I, Eiden RD, Nickerson AB, Molnar DS and Schuetze P (2020) Developmental cascades to children’s conduct problems: The role of prenatal substance use, socioeconomic adversity, maternal depression and sensitivity, and children’s conscience. *Development and Psychopathology* 32, 85–103. 30704548 10.1017/S095457941800144X PMC6675677

[ref29] Formánek T, Csajbók Z, Wolfová K, Kučera M, Tom S, Aarsland D and Cermakova P (2020) Trajectories of depressive symptoms and associated patterns of cognitive decline. *Scientific Reports* 10, 20888–20889. 33257789 10.1038/s41598-020-77866-6 PMC7705007

[ref30] Gariépy G, Honkaniemi H and Quesnel-Vallée A (2016) Social support and protection from depression: Systematic review of current findings in Western countries. *The British Journal of Psychiatry: The Journal of Mental Science* 209, 284–293. 27445355 10.1192/bjp.bp.115.169094

[ref31] Genova F, Neri E, Trombini E, Stella M and Agostini F (2022) Severity of preterm birth and perinatal depressive symptoms in mothers and fathers: Trajectories over the first postpartum year. *Journal of Affective Disorders* 298, 182–189. 34728282 10.1016/j.jad.2021.10.080

[ref32] Goodman R (1997) The Strengths and Difficulties Questionnaire: A research note. *Journal of Child Psychology and Psychiatry* 38, 581–586. 9255702 10.1111/j.1469-7610.1997.tb01545.x

[ref33] Guintivano J, Manuck T and Meltzer-Brody S (2018) Predictors of postpartum depression: A comprehensive review of the last decade of evidence. *Clinical Obstetrics & Gynecology* 61, 591–603. 29596076 10.1097/GRF.0000000000000368 PMC6059965

[ref34] Handelzalts JE, Ohayon S, Levy S and Peled Y (2024) Risk psychosocial factors associated with postpartum depression trajectories from birth to six months. *Social Psychiatry & Psychiatric Epidemiology* 59, 1685–1696. 38193942 10.1007/s00127-023-02604-y

[ref35] Hong L, Le T, Lu Y, Shi X, Xiang L, Liu M, Zhang W, Zhou M, Wang J, Xu D, Yu X and Zhao K (2022) Distinct trajectories of perinatal depression in Chinese women: Application of latent growth mixture modelling. *BMC Pregnancy & Childbirth* 22, 24–34. 35012496 10.1186/s12884-021-04316-0 PMC8751241

[ref36] Kiviruusu O, Pietikäinen JT, Kylliäinen A, Pölkki P, Saarenpää-Heikkilä O, Marttunen M, Paunio T and Paavonen EJ (2020) Trajectories of mothers’ and fathers’ depressive symptoms from pregnancy to 24 months postpartum. *Journal of Affective Disorders* 260, 629–637. 31542556 10.1016/j.jad.2019.09.038

[ref37] Levis B, Negeri Z, Sun Y, Benedetti A and Thombs BD (2020) Accuracy of the Edinburgh Postnatal Depression Scale (EPDS) for screening to detect major depression among pregnant and postpartum women: Systematic review and meta-analysis of individual participant data. *BMJ (British Medical Journal)* 371, m4022. 33177069 10.1136/bmj.m4022 PMC7656313

[ref38] Lim GY, Tam WW, Lu Y, Ho CS, Zhang MW and Ho RC (2018) Prevalence of depression in the community from 30 countries between 1994 and 2014. *Scientific Reports* 8, 2861–2871. 29434331 10.1038/s41598-018-21243-x PMC5809481

[ref39] Luo S (2017) Assortative mating and couple similarity: Patterns, mechanisms, and consequences. *Social and Personality Psychology Compass* 11, e12337.

[ref40] Matthey S, Barnett B, Kavanagh DJ and Howie P (2001) Validation of the Edinburgh Postnatal Depression Scale for men, and comparison of item endorsement with their partners. *Journal of Affective Disorders* 64, 175–184. 11313084 10.1016/s0165-0327(00)00236-6

[ref41] McCall-Hosenfeld JS, Phiri K, Schaefer E, Zhu J and Kjerulff K (2016) Trajectories of depressive symptoms throughout the peri- and postpartum period: Results from the first baby study. *Journal of Women’s Health* 25, 1112–1121. 10.1089/jwh.2015.5310 PMC5116682 27310295

[ref42] Molgora S, Fenaroli V, Malgaroli M and Saita E (2017) Trajectories of postpartum depression in Italian first-time fathers. *American Journal of Men’s Health* 11, 880–887. 10.1177/1557988316677692 PMC5675320 27885145

[ref43] Nieh HP, Chang CJ and Chou LT (2022) Differential trajectories of fathers’ postpartum depressed mood: A latent class growth analysis approach. *International Journal of Environmental Research & Public Health* 19, 1891–1901. 35162913 10.3390/ijerph19031891 PMC8835334

[ref44] Nordsletten AE, Larsson H, Crowley JJ, Almqvist C, Lichtenstein P and Mataix-Cols D (2016) Patterns of nonrandom mating within and across 11 major psychiatric disorders. *JAMA Psychiatry* 73, 354–361. 26913486 10.1001/jamapsychiatry.2015.3192 PMC5082975

[ref45] Parker G, Tupling H and Brown LB (1979) A Parental Bonding Instrument. *British Journal of Medical Psychology* 52, 1–10.

[ref46] Pietikäinen JT, Kiviruusu O, Kylliäinen A, Pölkki P, Saarenpää‐Heikkilä O, Paunio T and Paavonen EJ (2020) Maternal and paternal depressive symptoms and children’s emotional problems at the age of 2 and 5 years: a longitudinal study. *Journal of Child Psychology and Psychiatry* 61, 195–204. 31535379 10.1111/jcpp.13126

[ref47] Piler P, Kandrnal V, Kukla L, Andrýsková L, Švancara J, Jarkovský J, Dušek L, Pikhart H, Bobák M and Klánová J (2016) Cohort profile: The European Longitudinal Study of Pregnancy and Childhood (ELSPAC) in the Czech Republic. *International Journal of Epidemiology* 46, 1379–1379f. 10.1093/ije/dyw091 PMC5837270 27380795

[ref48] Rao WW, Zhu XM, Zong QQ, Zhang Q, Hall BJ, Ungvari GS and Xiang YT (2020) Prevalence of prenatal and postpartum depression in fathers: A comprehensive meta-analysis of observational surveys. *Journal of Affective Disorders* 263, 491–499. 31757623 10.1016/j.jad.2019.10.030

[ref49] Serhan N, Ege E, Ayrancı U and Kosgeroglu N (2013) Prevalence of postpartum depression in mothers and fathers and its correlates. *Journal of Clinical Nursing* 22, 279–284. 23216556 10.1111/j.1365-2702.2012.04281.x

[ref50] Shorey S, Chee CYI, Ng ED, Chan YH, Tam WWS and Chong YS (2018) Prevalence and incidence of postpartum depression among healthy mothers: A systematic review and meta-analysis. *Journal of Psychiatric Research* 104, 235–248. 30114665 10.1016/j.jpsychires.2018.08.001

[ref51] Tebeka S, Strat YL and Dubertret C (2016) Developmental trajectories of pregnant and postpartum depression in an epidemiologic survey. *Journal of Affective Disorders* 203, 62–68. 27280964 10.1016/j.jad.2016.05.058

[ref52] Trivers R (1972) Parental investment and sexual selection. In Campbell B edited by, *Sexual Selection and the Descent of Man, 1871–1971*. Chicago, IL: Aldine de Gruyter, 136–179.

[ref53] Volling BL, Yu T, Gonzalez R, Tengelitsch E and Stevenson MM (2019) Maternal and paternal trajectories of depressive symptoms predict family risk and children’s emotional and behavioral problems after the birth of a sibling. *Development and Psychopathology* 31, 1307–1324. 30394259 10.1017/S0954579418000743 PMC6500778

[ref54] Wang D, Li YL, Qiu D and Xiao SY (2021) Factors influencing paternal postpartum depression: A systematic review and meta-analysis. *Journal of Affective Disorders* 293, 51–63. 34171611 10.1016/j.jad.2021.05.088

[ref55] Whisman MA (2007) Marital distress and DSM-IV psychiatric disorders in a population-based national survey. *Journal of Abnormal Psychology* 116, 638–643. 17696721 10.1037/0021-843X.116.3.638

[ref56] Whisman MA and Bruce ML (1999) Marital dissatisfaction and incidence of major depressive episode in a community sample. *Journal of Abnormal Psychology* 108, 674–678. 10609431 10.1037//0021-843x.108.4.674

[ref57] Yang K, Wu J and Chen X (2022) Risk factors of perinatal depression in women: A systematic review and meta-analysis. *BMC Psychiatry* 22, 63–74. 35086502 10.1186/s12888-021-03684-3 PMC8793194

