# Supplementary material for: Trajectories of depressive symptoms of mothers and fathers over 11 years
Source: Epidemiol Psychiatr Sci. 2025 Apr 10;34:e23. doi: 10.1017/S2045796025000174 (PMC12037347; doi:10.1017/S2045796025000174)
Supplement: Csajbók et al. supplementary material [file S2045796025000174sup001.pdf]

Trajectories of depressive symptoms of mothers and fathers over 11 years

Zsófia Csajbók[a,*], Jakub Fořt[a,b], & Pavla Brennan Kearns[c]

[a] Department of Psychology and Life sciences, Faculty of Humanities, Charles University, Prague, Czech Republic
[b] Department of Zoology, Faculty of Science, Charles University, Prague, Czech Republic
[c] Department of Epidemiology, Second Faculty of Medicine, Charles University, Prague

*Corresponding author: Zsófia Csajbók, Ph.D., Faculty of Humanities, Charles University, Pátkova 5, Prague 8, 182 00, Czech Republic.
Email: zsofia.csajbok@fhs.cuni.cz

SUPPLEMENTARY INFORMATION

Supplementary Measures

Relationship maintenance was categorized in multiple ways. Couples were assigned an "unstable relationship" status if they changed their relationship status at any point during the study. Those labeled as "only married" had no relationship status recorded other than their first marriage. Couples were considered "continuously married" if they had at least two confirmed reports of being married (first marriage) with no indication of separation, divorce, or bereavement. The "married ever" category included couples married at any point during the study, while "married later" applied to those who began the study single and married afterward. Additional codes included: "other than first marriage" (remarriage), "bereavement," "separated," "divorced," "single" (at any point during the study), and "continuously cohabitated" for couples with no indication of living apart.

Demographic data included age of the parents, child's sex (male, female), town (Brno, Znojmo), and education (1 = *primary* to 8 = *postgraduate education*). Data on *socioeconomic resources* concerned income (monthly household income), crowding (number of people living in the household / number of rooms in the household), deprivation (how difficult it is to secure

basic things, such as food, clothes, rent, heating; higher values indicate greater deprivation), father's employment (yes, no), financial help (if parents or other relatives help out financially; yes, no), and living in own house (own house vs. with parents or other). We also included information about social network (sum of 10 items assessing the quality and the quantity of the relationships, where higher values indicate a greater network) and social support (sum of 10 items, where higher values indicate more social support). Further information was reported by the mother of the child at 6 months on childcare provided by other people (father, other family members, someone who is not family) expressed in hours per week and the age of the child (in months) when they started to take care of the child.

Information about *emotional life* concerned relationship aggression (how aggressive is the relationship with the partner, higher score indicates more aggression), affection (how affectionate is the relationship with the partner, higher score indicates more affection), and love of the baby (how long it took the mother to love the baby; immediately, shortly, over a week, don't love them yet). Information about *health* concerned the number of diseases (sum of current 25 comorbidities), substance use (use of marijuana, psychostimulants, sleeping pills, crack, cocaine, or heroin; yes, no), smoking (non-smoker, current smoker), alcohol use (days in the past month consuming the equivalent of two beers, 0.5 l wine, four shots; 0 = *not one day* to 5 = *every day*), use of psychotropic pills (antidepressants, sedatives, or sleeping pills; 1 = *not at all* to 4 = *daily*). *Obstetric history* included data about previous pregnancy (yes, no), number of previous pregnancies (continuous), number of own children (continuous), history of miscarriage (yes, no), number of miscarriages (continuous), history of abortion (yes, no), number of abortions (continuous), and obstetric treatment (undergone any treatment for getting pregnant; yes, no).

Information about *parental history* includes stressful life events (sum of 41 events since the woman got pregnant), parental care (derived from items of the Parental Bonding Instrument,

PBI, Parker et al., 1979, measuring how caring their mother was, with higher scores indicating more care), overprotection (derived from the PBI items measuring how overprotective their mother was, with higher scores indicating more overprotection), home stability (if their parents were predictable; 1 = *never*, 4 = *always*), and sexual abuse (if they experienced sexual abuse during childhood or adolescence; never, once, more than once).

Offspring temperament was assessed in the newborn questionnaire, in which parents rated 14 items on a Likert scale (1 = *not at all*, 4 = *very much*) based on how much the child shows the characteristics of being whiny, cranky, satisfied, etc. *Offspring mental health* was completed by the child and included internalizing and externalizing symptoms derived from the Strengths and Difficulties Questionnaire (where higher score indicates more symptoms, Goodman, 1997), satisfaction with life (a 5-item scale designed to measure global cognitive judgments of one's life satisfaction, higher score indicates more satisfaction, Diener et al., 1985), and stressful life events (average of the ratings of experience of stressful events and how upset they made the respondent on a 0 = *it did not happen* to 4 = *it happened and made me very upset* scale; year 11: 20 events, year 15: 28 events, year 18 and 19: 33 events).

Supplementary Data analysis

Latent trajectory identification

Models were tested with Mplus version 8.11. Since the software handles data variance best if kept below 10, we divided the total Edinburgh Postnatal Depressive Symptoms (EPDS) scores by three. This did not influence the longitudinal patterns of the depression scores, only facilitated the model estimation. Thus, after the analyses were performed, we plotted the data and reported descriptive statistics using the original scoring of the questionnaire. Missing data was handled with Full Information Maximum Likelihood estimation method.

First, the dyadic parallel growth process was investigated with various growth models in the overall sample. We fitted against the maternal and paternal depression scores a latent

growth model assuming linear growth (i.e., latent intercept and slope factors), a latent curved growth model assuming curved growth (i.e., latent intercept, slope, and curve factors), and a latent base growth model that allows for freely estimated patterns. The maternal and paternal latent growth factors were all correlated. Maximum likelihood robust estimator was used, because the distribution of fathers' depression scores was leptokurtic (Table S1). We relied on the root mean square error of approximation (RMSEA) and the standard root-mean-square residual (SRMR) as model fit indices and considered them indicating acceptable model fit if lower than 0.08; and the comparative fit index (CFI) and Tucker-Lewis index (TLI) if higher than 0.90 (Brown, 2006). The latent growth model yielded acceptable model fit, while the latent curve growth model did not converge. The best model fit was obtained with the latent base growth model, thus we submitted this to the mixture modeling (Table S2).

We employed three different kinds of growth mixture models, to inspect their relative utility on our data. Mixture modeling is a technique that can identify homogeneous subgroups in our data which are sufficiently similar to each other within classes and dissimilar across classes. The growth aspect of it can unfold longitudinal patterns among the participants, grouping together those who have similar longitudinal trajectories to each other with high probability. The three methods employed for this analysis have slight alterations between themselves. All models estimate a latent class (i.e., an unobserved latent categorical variable), and each latent class has their own values estimated as latent growth factors. The latent growth factors predict the various longitudinal growth trajectories of the observed repeated measures variables. In the latent class growth model (LCGM), the variances of the latent growth factors are fixed at zero, to facilitate model estimation. In the growth mixture modeling (GMM) framework each class has estimated variances of the latent growth factors, but they are fixed to be equal across classes. In the covariance pattern growth mixture model (CPGMM; McNeish & Harring, 2020) the observed variables are correlated and the covariances are estimated

uniquely in each class (i.e., they are not fixed at zero, neither to be equal across classes). We performed the analyses following the recommended guidelines (Van De Schoot et al., 2017).

The model selection was performed relying on a range of criteria. We utilized the Akaike Information Criterion (AIC; Akaike, 1973), Bayesian Information Criterion (BIC; Schwartz, 1978), entropy, the Vuong-Lo-Mendell-Rubin Likelihood Ratio Test (VLMR LRT; Vuong, 1989), the Lo-Mendell-Rubin Adjusted Likelihood ratio test (LMR ALRT; Lo et al, 2001), and the parametric bootstrapped likelihood ratio test (BLRT; McLachlan & Peel, 2004). Preferably, the best model should have the lowest AIC and BIC among all the tested 2-, 3-, 4-, 5-, and 6-class solutions; entropy larger than .70 or preferably .80; and significant VLMRT LRT, LMR ALRT, and BLRT tests. All these model criteria would indicate that extracting k + 1 class describe the data better than extracting k class (starting with k = 1, i.e., assuming the sample is homogeneous). We also considered model interpretability and size of the smallest extracted class. If the resulting longitudinal trajectories were not essentially different (e.g., all classes had essentially the same slopes with different intercepts), we rejected the models. We also rejected the models if one of the resulting classes was too small (< 5%), because that could indicate that the ill-fitting participants were discarded there.

The growth mixture model (holding the variances equal between the classes) yielded interpretatively equivalent classes, that is, the slopes were essentially the same (and zero, i.e., the trajectories were constant and did not grow or decrease over time) among all the classes. The 3-class model already obtained a class with only a 2% membership, and the 4- and 5-class solutions were similar and did not improve, even though the VLMRT LRT and LMR ALRT tests supported the extraction of four classes (but not five). On the other hand, while the CPGMM model was recommended (McNeish et al., 2023) to use for it possibly being able to avoid artefacts, the solutions were not distinctive, that is, the class intercepts were very similar to each other. Therefore, we selected the LCGM model to rely on.

The 2-, 3-, 4-, 5-, and 6-class LCGM solutions were all replicated with at least three sets of random starts. The best loglikelihood values were always replicated multiple times. The fourth model run was performed to obtain the parametric BLRT test using the LRT-starts option. We chose for final model the 5-class model, because that model had lower AIC and BIC (i.e., the most widely considered and most reliable model selection indicator in growth mixture methods, Van De Schoot et al., 2017) than the 4-class model, it had an acceptable entropy (.750), and significant parametric BLRT test, even though the VLMRT LRT and LMR ALRT tests were not significant (Table S3). Since the 5-class solution did not have problematic characteristics, such as convergence problems, interpretability problems, or small class sample sizes, but it was qualitatively different from the 4-class solution, and the most preferred model indicator (as well as the BLRT test) preferred the k+1 solution, and our large sample size also allowed for a more nuanced grouping, we opted for the more complex, 5-class solution. Relying on the 5-class solution over the 4-class solution, we aimed to discover more subtle differences between different couple-constellations. On the other hand, we decided against the 6-class model, even though it yielded even smaller AIC and BIC than the 5-class model, because it already extracted a class with a 2.6% membership rate, and the extraction of one more class above 5 did not provide a parsimonious additional interpretative value (Figures S1a-f).

Eventually, we performed a sensitivity analysis to inspect whether the relatively large missingness in the data influenced the results we obtained on the overall sample. We performed the same classification analysis on a subset of participants who had very good data coverage (both mother and father had at least 6 time-points out of 8, n = 2528); and on a subset of participants who had full data coverage (both mother and father had all 8 data points covered, n = 951). The yielded latent trajectory patterns were essentially equivalent to the patterns obtained on the original, full sample (Supplementary Figures S2a-b).

The 5-class solution we selected for further investigation and final latent trajectory model can be seen in Table S4. The mean intercept and slope latent growth factors estimated in the 5-class model are also shown in Table S4. The estimated loadings of the observed variables on the latent slope factors are in Table S5. The 5-class model showed the following patterns of dyads: Class 1) mother has elevated depression, father is non-depressed (24.19%), Class 2) both mother and father have elevated depression (19.61%), Class 3) both mother and father are constantly non-depressed (42.30%), Class 4) both mother and father are constantly depressed (4.93%), and Class 5) mother is constantly depressed, father has elevated depression (8.97%), see Figure 1.

Supplementary Results

The covariates selected for reporting in this supplementary text were selected based on effect size (i.e., |r| > .10 and |d| > 0.20 at least at one timepoint in one parent). Testing covariates of depressive symptoms on the overall sample was performed in two sections. First, binary variables were compared with independent samples *t* tests (reported in *d*s). Among the relationship maintenance covariates, being in an unstable relationship, separated, divorced, and single were associated with having more depressive symptoms in both fathers and mothers (Table S7). Bereavement did not influence fathers, but mothers at 7 and 11 years of the child. Being married later influenced fathers prenatally, but not mothers. Being separated had the strongest negative influence on both mothers' and fathers' depressive symptoms (second strongest covariate overall for fathers, averaged *d*s = -0.38; strongest covariate overall for mothers, averaged *d*s = -0.40), while being married ever had the strongest positive influence (fathers' average *d*s = 0.18, mothers' average *d*s = 0.24) among the relationship maintenance variables, but overall as well.

Among socioeconomic resources, being employed had a stronger positive influence on fathers (especially postnatally) than on mothers. Mothers had fewer depressive symptoms

prenatally and fathers postnatally if they utilized obstetric treatment to get pregnant. Fathers and mothers who used psychoactive substances (strongest covariate overall for fathers, averaged *d*s = -0.58; second strongest covariate overall for mothers, averaged *d*s = -0.30) and were current smokers had more depressive symptoms essentially throughout the study. Depressive symptoms thus did not meaningfully change with not being in first marriage, partner's care for the baby, town, child's sex, living in own house, needing financial help, previous pregnancy, having a miscarriage, or abortion.

Next, continuous variables were correlated with depressive symptoms in the overall sample. Parents experiencing deprivation had more depressive symptoms, particularly mothers (Table S8). Having a larger social network and more social support was associated with lower depressive symptoms in both fathers and mothers. More aggression and less affection were associated with more depressive symptoms in fathers and particularly in mothers. Experiencing more overprotection during childhood and more stressful life events correlated with more depressive symptoms in both fathers and mothers. In both parents, more diseases and taking psychotropic pills more often was associated with more depressive symptoms. Having a whiny, cranky baby was associated with more symptoms and a satisfied baby with less depressive symptoms in fathers till 6 months and particularly in mothers throughout the entire study period. Therefore, in the overall sample, depressive symptoms did not meaningfully correlate with age, income, crowding, education, love for the baby, alcohol use, and help received from the partner, family, or non-family. Parents' childhood experiences of parental care, home stability, and sexual abuse did not correlate with depressive symptoms. Obstetric history, such as the number of previous pregnancies, own children, miscarriages, and abortion did not correlate with depressive symptoms.

Lastly, we also correlated offspring internalizing and externalizing symptoms with paternal and maternal depressive symptoms at the age of 11 years of the child. Parents'

depressive symptoms at 11 years weakly correlated with the child's internalizing problems at 11 years (father: r = .10, p < .001, mother: r = .09, p < .001) and even more weakly with externalizing problems at 11 years (father: r = .06, p < .001, mother: r = .05, p < .001). Parents' depressive symptoms at age 11 correlated with offspring internalizing and externalizing problems at the age of 15 and 18 too (rs ranging between .05 and .13 in fathers and .06 and .11 in mothers, Table S9). Offspring's satisfaction with life at the age of 15 years (but not at age 19) negatively correlated with parents' depressive symptoms at age 11 (father: r = -.09, p < .001, mother: r = -.10, p < .001). Offspring experienced more stressful life events at ages 11, 15, 18, and 19 years if their parents had more depressive symptoms at age 11 (rs ranging from .04 to .18 in fathers, and from .07 to .11 in mothers, Table S9). Parental depressive symptoms did not correlate with offspring self-esteem.

Supplementary Discussion

We studied the risk factors of perinatal depressive symptoms on a prenatal cohort sample of new parents. On the overall sample, we found that relationship maintenance (e.g., unstable relationship, being married, or separated) were associated with depressive symptoms with small to medium effect sizes. Socioeconomic resources (e.g., employment and social support), health (particularly substance and psychotropic pill usage), emotional life, parental history, and offspring temperament were weakly correlated with depressive symptoms of both mothers and fathers. Offspring mental health in adolescent age was also weakly correlated with parental depressive symptoms.

When comparing the results obtained on the overall sample versus comparing the five classes (see in the main text), we can see that relationship maintenance (particularly being separated) was a robust covariate in both analyses. Socioeconomic resources and emotional life also showed articulated results in both the overall sample and across the classes, with comparable relative effect sizes among all covariates (i.e., the effects were demonstrably there,

but not among the strongest effect sizes). Health, particularly psychotropic pill usage, was demonstrably correlated with depressive symptoms in both analyses. On the other hand, obstetric history had a stronger association with the classes than with the overall sample. Also, while overprotection in the parents' childhood was associated with depressive symptoms overall, it did not differ much between the classes. Stressful life events of parents were correlated in both analyses, however. More characteristics of the child's temperament were associated with the overall scores than with the class memberships. Offspring mental health outcomes were slightly more consistently associated with the overall scores than with the class memberships. Otherwise, the effect sizes were comparable, generally weak to moderate, across the two types of analysis (i.e., when performed on the overall sample versus across the classes).

Table S1. Descriptive statistics and data coverage of the depression scores

	Edinburgh Postnatal Depression Scale	*N*	Cronbach's alpha	Min.	Max.	Mean	*SD*	Skewness	Kurtosis
Father	prenatal	4112	0.76	0.00	30.00	4.22	3.61	1.33	2.72
	newborn	4517	0.77	0.00	30.00	4.16	3.62	1.37	2.95
	6 months	4101	0.78	0.00	28.00	3.96	3.53	1.34	2.51
	18 months	3104	0.81	0.00	26.00	4.31	3.80	1.43	2.67
	3 years	3090	0.81	0.00	23.00	4.26	3.72	1.27	1.85
	5 years	2941	0.83	0.00	30.00	4.17	3.93	1.51	3.10
	7 years	2566	0.83	0.00	29.00	4.28	3.96	1.51	3.08
	11 years	1918	0.81	0.00	26.67	6.29	3.97	1.36	2.35
Mother	prenatal	4347	0.81	0.00	29.00	6.49	4.49	0.81	0.64
	newborn	4787	0.82	0.00	29.00	6.63	4.53	0.84	0.75
	6 months	4366	0.83	0.00	27.00	6.13	4.31	0.84	0.57
	18 months	3434	0.84	0.00	25.00	6.18	4.50	0.90	0.66
	3 years	3516	0.85	0.00	26.00	6.51	4.63	0.91	0.82
	5 years	3432	0.87	0.00	30.00	6.14	4.69	0.97	0.88
	7 years	3121	0.86	0.00	28.00	6.37	4.84	1.01	0.96
	11 years	2460	0.85	0.00	27.14	8.09	4.81	0.88	0.54

Note. Min. = minimum. Max. = maximum. *SD* = standard deviation.

Table S2. Model fits of the latent growth model with fixed linear slopes; and the latent base growth model with freed slopes on the total sample

Model	Estimated	Shape	χ^2(df)	RMSEA	CFI	TLI	SRMR	AIC	BIC
latent growth	intercept, slope	linear	1454.177 (122)	0.044 (.042, .047)	0.909	0.911	0.057	176836.266	177034.739
latent base growth	intercept, slope	free	1166.173 (110)	0.042 (.040, .044)	0.928	0.922	0.043	176489.695	176767.557

Note. RMSEA=root mean square of approximation; CFI=comparative fit index; TLI=Tucker-Lewis index; AIC=Akaike information criterion; BIC=Bayesian information criterion.

Table S3. Model parameters and results of the 2-, 3-, 4-, 5-, and 6-class latent base class growth models

N of classes	Best Loglikelihood	AIC	BIC	Entropy	VLM RLR	LMR ALRT	BLRT
2	-92370.830	184815.661	185060.444	.778	$p < .001$	$p < .001$	$p < .001$
3	-90639.667	181363.333	181641.196	.793	$p < .001$	$p < .001$	$p < .001$
4	-89462.264	179018.529	179329.470	.773	$p < .001$	$p < .001$	$p < .001$
5	-88856.637	177817.274	178161.294	.750	$p = .104$	$p = .112$	$p < .001$
6	-88487.597	177089.195	177466.294	.757	$p = .612$	$p = .614$	$p < .001$

Note. AIC=Akaike information criterion; BIC=Bayesian information criterion; VLM RLT=Vuong-Lo-Mendell-Rubin likelihood ratio test; LMR ALRT=Luo-Mendell-Rubin adjusted likelihood ratio test; BLRT=bootstrap likelihood ratio test.

Table S4. Class proportions and mean intercept and slope results in the 5-class dyadic latent base growth model

	N of class members	% of total *N*	Mean maternal latent intercept factor	Mean maternal latent slope factor	Mean paternal latent intercept factor	Mean paternal latent slope factor
Class 1	1335	24.19%	2.746***	0.513***	1.068***	0.672***
Class 2	1082	19.61%	2.077***	0.560***	2.298***	0.825***
Class 3	2334	42.30%	1.264***	0.551***	0.848***	0.699***
Class 4	272	4.93%	3.606***	0.276	3.925***	0.615*
Class 5	495	8.97%	4.272***	0.356*	1.848***	0.734***

Note. Variables in the analyses were divided by three to aid model convergence (following the common practice using Mplus software). This means that the true intercepts and slopes performed on the original scores would be three times the intercepts and slopes received in this analysis. This is why we presented the intercepts and slopes in Table 1 in converted (i.e., 3 times the scores presented here), original scaling.

* $p < .05$. *** $p < .001$

Table S5. Estimated loadings of the depressive symptom measures at each time point on the latent slope factors in the 5-class latent basis class growth model

Postnatal depression	Estimated loadings of mothers (*SEM*)	Estimated loadings of fathers (*SEM*)
prenatal	0.000 (0.000)	0.000 (0.000)
newborn	0.042 (0.046)	-0.057 (0.029)
6 months	-0.285 (0.057)***	-0.138 (0.033)***
18 months	-0.252 (0.062)***	0.047 (0.035)
3 years	-0.008 (0.057)	0.052 (0.034)
5 years	-0.255 (0.065)***	0.002 (0.040)
7 years	-0.111 (0.064)	0.074 (0.040)
11 years	1.000 (0.000)	1.000 (0.000)

SEM = standard error of the mean.
*** $p < .001$

Table S6. Descriptive statistics and data coverage of all covariates

	N	Min.	Max.	Mean	*SD*
Relationship maintenance					
Unstable relationship	5515	0.00	1.00	0.14	0.35
Continuously married	5518	0.00	1.00	0.52	0.50
Only married	5518	0.00	1.00	0.65	0.48
Married ever	5343	0.00	1.00	0.92	0.28
Not first marriage	5343	0.00	1.00	0.16	0.37
Bereavement	5346	0.00	1.00	0.02	0.13
Separated	5343	0.00	1.00	0.05	0.22
Divorced	5343	0.00	1.00	0.10	0.30
Married later	5170	0.00	1.00	0.05	0.22
Single ever	5343	0.00	1.00	0.10	0.30
Continuous cohabitation	5339	0.00	1.00	0.90	0.30
Demographic data					
Age (m.)	4385	15.00	48.00	24.34	4.76
Age (f.)	4347	15.00	58.00	27.40	5.87
Education (m.)	4413	1.00	8.00	3.86	2.00
Education (f.)	4419	1.00	8.00	3.82	2.18
Town (Brno)	5518	0.00	1.00	0.75	0.43
Child’s sex (female)	5514	0.00	1.00	0.48	0.50
Socioeconomic resources					
Deprivation	4267	5.00	20.00	8.05	3.22
Employed (f.)	4449	0.00	1.00	0.92	0.27
Social network (m.)	4388	2.00	30.00	21.25	4.08
Social network (f.)	4241	2.00	30.00	21.44	4.25
Social support (m.)	4251	3.00	28.00	17.41	4.08
Social support (f.)	4088	4.00	30.00	19.30	4.49
Living in own house	4363	0.00	1.00	0.59	0.49
Financial help	2823	0.00	1.00	0.31	0.46
Income	2735	1500.00	60000.00	5586.72	2882.32
Crowding	4090	0.00	13.00	1.62	1.00
Partner taking care of baby	4100	0.00	1.00	0.77	0.42
Partner taking care (hw)	2458	1.00	75.00	17.05	14.66
Partner taking care (mth)	2677	0.00	6.00	1.28	1.42
Family taking care (hw)	1433	1.00	75.00	9.89	11.70
Family taking care (month)	1444	0.00	6.00	1.86	1.63
Non-family taking care (hw)	131	1.00	42.00	5.33	6.57
Non-family taking care (mth)	121	0.00	6.00	2.96	1.94
Emotional life					
Aggression (m.)	4338	3.00	15.00	7.39	1.84
Aggression (f.)	4229	3.00	15.00	7.43	1.92
Affection (m.)	4339	6.00	30.00	23.86	3.67
Affection (f.)	4230	6.00	30.00	24.08	3.64
Love of the baby	4507	1.00	4.00	1.33	0.59
Parental history					
Stressful life events (m.)	4269	0.00	41.00	2.98	2.79
Stressful life events (f.)	4107	0.00	40.00	3.07	2.80
Parental care (m.)	4121	13.00	48.00	39.21	6.34
Parental care (f.)	3964	12.00	48.00	38.75	5.76
Overprotection (m.)	4103	10.00	40.00	22.33	4.67
Overprotection (f.)	3957	10.00	40.00	22.78	4.76
Home stability (m.)	4280	1.00	4.00	2.91	0.53
Home stability (f.)	4106	1.00	4.00	2.92	0.58
Sexual abuse (m.)	4085	0.00	2.00	0.35	0.63

Sexual abuse (f.)	3898	0.00	2.00	0.13	0.42
Health					
N of diseases (m.)	5266	0.00	7.67	0.72	1.04
N of diseases (f.)	4130	0.00	16.70	0.67	1.04
Substance use (m.)	4282	0.00	1.00	0.02	0.14
Substance use (f.)	5277	0.00	1.00	0.03	0.17
Smoking (m.)	4824	0.00	1.00	0.17	0.37
Smoking (f.)	4165	0.00	1.00	0.45	0.50
Psychotropic pills (m.)	5518	1.00	4.00	1.05	0.27
Psychotropic pills (f.)	5518	1.00	4.00	1.04	0.23
Alcohol use (m.)	4017	0.00	5.00	0.15	0.65
Alcohol use (f.)	5254	0.00	5.00	1.61	1.34
Obstetric history					
Previous pregnancy	4372	0.00	1.00	0.62	0.49
Miscarriage	2705	0.00	1.00	0.24	0.43
Abortion	2705	0.00	1.00	0.36	0.48
Obs. treatment	412	0.00	1.00	0.34	0.48
N of previous pregnancies	2707	1.00	9.00	1.81	1.16
N of children	2666	0.00	8.00	1.03	0.74
N of miscarriage	652	1.00	5.00	1.18	0.48
N of abortion	975	1.00	5.00	1.31	0.63
Offspring mental health					
Stressful life events (11y)	2424	0.00	3.50	0.44	0.36
Stressful life events (15y)	1557	0.00	3.50	0.43	0.34
Stressful life events (18y)	570	0.00	1.64	0.46	0.28
Stressful life events (19y)	533	0.00	2.33	0.35	0.30
SDQ: internalizing[1] (11y)	2319	0.00	26.33	7.98	4.09
SDQ: externalizing[1] (11y)	2320	0.00	28.75	9.43	4.61
SDQ: internalizing[2] (15y)	1568	0.00	18.00	5.28	3.27
SDQ: externalizing[2] (15y)	1568	0.00	17.00	6.57	3.41
SDQ: internalizing[2] (18y)	596	0.00	17.00	5.55	3.20
SDQ: externalizing[2] (18y)	596	0.00	17.00	6.17	3.35
Self-esteem (15y)	1532	13.00	40.00	29.45	4.49
Self-esteem (18y)	585	14.00	40.00	30.46	4.88
Self-esteem (19y)	333	11.00	40.00	31.16	5.56
Satisfaction with life (15y)	1545	5.00	25.00	17.58	3.96
Satisfaction with life (19y)	273	5.00	25.00	17.91	4.47
Offspring temperament					
Cranky	4340	1.00	4.00	1.57	0.66
Calm	4461	1.00	4.00	3.22	0.76
Chatty	4301	1.00	4.00	2.85	0.92
Whiny	4358	1.00	4.00	1.80	0.75
Demanding	4362	1.00	4.00	2.41	0.97
Angry	4340	1.00	4.00	1.54	0.72
Clingy	4369	1.00	4.00	3.33	0.67
Lively	4380	1.00	4.00	3.53	0.61
Social	4257	1.00	4.00	3.17	0.85
Closed	4225	1.00	4.00	1.38	0.56
Stubborn	4221	1.00	4.00	1.60	0.77
No interest	4256	1.00	4.00	1.31	0.58
Satisfied	4355	1.00	4.00	3.43	0.62
Bright	4300	1.00	4.00	3.40	0.64

Note. m = mother. f = father. *M* = mean. Min. = minimum. Max. = maximum. *SD* = standard deviation.

[1] SDQ items were measured on a 0 to 3 scale.

[2] SDQ items were measured on a 0 to 2 scale.

Cronbach's alphas:

Stressful life events (11y) = 0.65.
Stressful life events (15y) = 0.77.
Stressful life events (18y) = 0.73.
Stressful life events (19y) = 0.80.
SDQ: internalizing[1] (11y) = 0.66.
SDQ: externalizing[1] (11y) = 0.76.
SDQ: internalizing[2] (15y) = 0.69.
SDQ: externalizing[2] (15y) = 0.68.
SDQ: internalizing[2] (18y) = 0.71.
SDQ: externalizing[2] (18y) = 0.70.
Self-esteem (15y) = 0.83.
Self-esteem (18y) = 0.87.
Self-esteem (19y) = 0.90.
Satisfaction with life (15y) = 0.80.
Satisfaction with life (19y) = 0.87.

Table S7. Independent samples *t* tests comparing father's and mother's depressive symptoms over time across binary characteristics (Cohen's *d*)

Father	prenatal	newborn	6 months	18 months	3 years	5 years	7 years	11 years	average
Relationship maintenance									
Unstable relationship	-0.20***	-0.22***	-0.20***	-0.24***	-0.23***	-0.14*	-0.29***	-0.04	-0.20
Continuously married	0.13***	0.22***	0.17***	0.21***	0.13**	0.12**	0.12*	0.02	0.14
Only married	0.16***	0.23***	0.14***	0.2***	0.12**	0.11**	0.13**	0.02	0.14
Married ever	0.21**	0.24***	0.18*	0.38***	0.16	0.23*	0.12	-0.11	0.18
Married later	-0.23**	-0.09	-0.08	-0.04	0.02	0.06	-0.02	0.09	-0.04
Bereavement	0.04	-0.13	< 0.01	0.08	0.02	0.05	-0.08	-0.01	< 0.01
Separated	-0.30**	-0.41***	-0.35***	-0.39***	-0.45***	-0.37**	-0.69***	-0.09	-0.38
Divorced	-0.21***	-0.24***	-0.20***	-0.29***	-0.28***	-0.28**	-0.33**	-0.02	-0.23
Singlehood	-0.25***	-0.11*	-0.13*	-0.16*	-0.08	0.01	-0.08	0.1	-0.09
Continuous cohabitation	0.31***	0.29***	0.21***	0.25***	0.17*	0.04	0.08	-0.12	0.15
Socioeconomic resources									
Employed (f.)	0.18*	0.30***	0.21**	0.06	0.14	0.12	0.08	0.06	0.14
Health & Obstetric history									
Obs. treatment	0.16	0.30**	0.05	0.23	0.14	0.04	0.2	0.27	0.17
Substance use (f.)	-0.77***	-0.76***	-0.70***	-0.62***	-0.48***	-0.46**	-0.37**	-0.47*	-0.58
Smoking (f.)	-0.04	-0.05	-0.11**	-0.08	-0.11*	-0.09*	-0.11*	-0.17**	-0.10
**Mother**	**prenatal**	**newborn**	**6 months**	**18 months**	**3 years**	**5 years**	**7 years**	**11 years**	**average**
Relationship maintenance									
Unstable relationship	-0.16***	-0.15***	-0.15***	-0.30***	-0.22***	-0.25***	-0.24***	-0.05	-0.19
Continuously married	0.14***	0.11***	0.13***	0.25***	0.20***	0.19***	0.21***	0.11**	0.17
Only married	0.16***	0.11***	0.11**	0.25***	0.19***	0.18***	0.2***	0.1*	0.16
Married ever	0.33***	0.16**	0.19**	0.31***	0.27**	0.25**	0.16	0.27**	0.24
Married later	-0.14	-0.03	0.06	-0.05	0.03	-0.01	0.04	0.08	< 0.01
Bereavement	-0.10	-0.15	-0.10	-0.12	-0.16	-0.16	-0.26*	-0.32*	-0.17
Separated	-0.34***	-0.41***	-0.36***	-0.50***	-0.47***	-0.46***	-0.43***	-0.21*	-0.40
Divorced	-0.21***	-0.15**	-0.13*	-0.31***	-0.25***	-0.24***	-0.25***	-0.08	-0.20
Singlehood	-0.25***	0.01	-0.05	-0.19**	-0.09	-0.06	< 0.01	0.01	-0.08
Continuous cohabitation	0.27***	0.18**	0.15**	0.28***	0.23***	0.18**	0.19**	0.03	0.19
Socioeconomic resources									
Employed (f.)	0.23**	0.03	0.05	-0.04	0.12	0.14	0.03	0.06	0.08
Health & Obstetric history									
Obs. treatment	0.22*	0.12	0.02	-0.01	0.02	0.03	0.05	0.12	0.07
Substance use (m.)	-0.42**	-0.29*	-0.33*	-0.47*	-0.28	-0.36*	0.08	-0.34	-0.30
Smoking (m.)	-0.32***	-0.12**	-0.12*	-0.18***	-0.11*	-0.21***	-0.27***	-0.16*	-0.19

Note. Numbers presented are Cohen's *d* effect sizes. Negative *d*s indicate that the presented variables are associated with more depressive symptoms, positive *d*s indicate that the variables are associated with less depressive symptoms. m = mother. f = father. * $p < .05$. ** $p < .01$. *** $p < .001$.

Table S8. Correlations between parents' depressive symptoms over time and parental characteristics (Pearson's *r)*

Father	prenatal	newborn	6 months	18 months	3 years	5 years	7 years	11 years	average
Socioeconomic resources									
Deprivation	.09***	.07***	.09***	.08***	.08***	.08***	.08***	.08***	.08
Social network (f.)	-.15***	-.15***	-.13***	-.14***	-.13***	-.13***	-.11***	-.09***	-.13
Social support (f.)	-.26***	-.22***	-.21***	-.23***	-.16***	-.23***	-.16***	-.13***	-.20
Emotional life									
Aggression (f.)	.19***	.18***	.18***	.16***	.13***	.14***	.13***	.13***	.16
Affection (f.)	-.19***	-.15***	-.14***	-.15***	-.13***	-.17***	-.14***	-.12***	-.15
Parental history									
Overprotection (f.)	.12***	.13***	.15***	.14***	.12***	.10***	.13***	.13***	.13
Stressful life events (f.)	.28***	.23***	.19***	.20***	.16***	.18***	.15***	.17***	.14
Health									
N of diseases (f.)	.17***	.12***	.12***	.13***	.14***	.14***	.17***	.16***	.13
Psychotropic pills (f.)	.11***	.11***	.21***	.15***	.13***	.11***	.10***	.09***	.20
Offspring temperament									
Whiny baby	.03	.06***	.05***	.04	.03	.04	.03	.02	.04
Cranky baby	.04*	.07***	.06***	.05*	.05*	.05*	.03	.03	.05
Satisfied baby	-.04*	-.05***	-.05***	-.03	-.03	< .01	-.01	.01	-.03
**Mother**	**prenatal**	**newborn**	**6 months**	**18 months**	**3 years**	**5 years**	**7 years**	**11 years**	**average**
Socioeconomic resources									
Deprivation	.15***	.15***	.19***	.16***	.16***	.18***	.17***	.14***	.16
Social network (m.)	-.13***	-.08***	-.11***	-.12***	-.13***	-.13***	-.09***	-.11***	-.11
Social support (m.)	-.26***	-.18***	-.19***	-.21***	-.18***	-.16***	-.16***	-.16***	-.19
Emotional life									
Aggression (m.)	.28***	.21***	.22***	.24***	.19***	.21***	.17***	.17***	.21
Affection (m.)	-.20***	-.19***	-.20***	-.17***	-.14***	-.17***	-.14***	-.12***	-.17
Parental history									
Overprotection (m.)	.13***	.13***	.09***	.12***	.12***	.13***	.12***	.09***	.12
Stressful life events (m.)	.34***	.25***	.21***	.23***	.24***	.22***	.23***	.19***	.11
Health									
N of diseases (m.)	.19***	.11***	.09***	.12***	.09***	.11***	.10***	.09***	.14
Psychotropic pills (m.)	.10***	.17***	.20***	.12***	.15***	.14***	.14***	.07***	.24
Offspring temperament									
Whiny baby	.11***	.14***	.11***	.11***	.11***	.11***	.07***	.08***	.11
Cranky baby	.12***	.17***	.14***	.14***	.12***	.11***	.09***	.12***	.13
Satisfied baby	-.07***	-.15***	-.12***	-.11***	-.09***	-.07***	-.08***	-.09***	-.10

Note. m = mother. f = father. *r* coefficients were Fisher's *z* transformed before averaging and back-transformed for reporting.
* $p < .05$. ** $p < .01$. *** $p < .001$.

Table S9. Correlations between parental depressive symptoms at the offspring's age of 11 years and offspring mental health

		Father	Mother
SDQ: internalizing symptoms (11y)	*r*	.10***	.09***
	n	1786	2253
SDQ: externalizing symptoms (11y)	*r*	.06*	.05*
	n	1782	2255
SDQ: internalizing symptoms (15y)	*r*	.09***	.11***
	n	1154	1443
SDQ: externalizing symptoms (15y)	*r*	.07*	.11***
	n	1154	1443
SDQ: internalizing symptoms (18y)	*r*	.13*	.09*
	n	453	548
SDQ: externalizing symptoms (18y)	*r*	.05	.06
	n	453	548
Satisfaction with life (15y)	*r*	-.09***	-.10***
	n	1136	1422
Satisfaction with life (19y)	*r*	-.13	-.05
	n	217	251
Stressful life events (11y)	*r*	.04	.11***
	n	1862	2355
Stressful life events (15y)	*r*	.09***	.11***
	n	1145	1434
Stressful life events (18y)	*r*	.18***	.07
	n	433	521
Stressful life events (19y)	*r*	.15***	.11*
	n	410	486

Note. y = years of age. SDQ = Strength and Difficulties Questionnaire.
* $p < .05$. ** $p < .01$. *** $p < .001$.

Table S10. Brown-Forsythe analysis of variance tests or chi-square tests comparing the five classes along parental covariates that had weak associations

	Class 1: Mother has elevated depression, father is non-depressed	Class 2: Both mother and father have elevated depression	Class 3: Both mother and father are constantly non-depressed	Class 4: Both mother and father are constantly depressed	Class 5: Mother is constantly depressed, father has elevated depression	η^2 or *V*
Relationship maintenance						
Married later *n*, %	63 (4.9%)	54 (5.3%)	116 (5.3%)	18 (7.4%)	25 (5.5%)	.02
Demographic data						
Age (m.) *M*, *SD*	24.22 (4.69)	24.56 (4.78)	24.02 (4.56)	25.58 (5.37)	25.21 (5.33)	.01***
Age (f.) *M*, *SD*	27.12 (5.65)	27.8 (6.08)	27.07 (5.65)	28.68 (6.85)	28.35 (6.4)	.01***
Education (m.) *M*, *SD*	3.84 (2.01)	3.92 (2.01)	3.88 (1.97)	3.82 (2.09)	3.69 (2.06)	<.01
Education (f.) *M*, *SD*	3.82 (2.15)	3.91 (2.26)	3.8 (2.15)	3.79 (2.33)	3.75 (2.18)	<.01
Town (Brno) *n*, %	1020 (76.4%)	824 (76.2%)	1746 (74.8%)	201 (73.9%)	367 (74.1%)	.02
Child's sex (female) *n*, %	647 (48.5%)	517 (47.8%)	1096 (47%)	137 (50.4%)	254 (51.3%)	.03
Socioeconomic resources						
Living in own house *n*, %	636 (58.6%)	502 (59.8%)	1120 (59.4%)	109 (57.1%)	217 (59.6%)	.01
Financial help *n*, %	216 (31.2%)	179 (32%)	354 (29.7%)	40 (32.8%)	96 (37.1%)	.04
Income *M*, *SD*	5452.53 (2070.9)	5443.55 (2105.19)	5760.1 (3546.78)	5756.06 (4007.13)	5393.78 (2091.09)	<.01
Crowding *M*, *SD*	1.65 (0.94)	1.61 (1.08)	1.58 (0.94)	1.84 (1.31)	1.7 (1.12)	<.01*
Partner taking care of baby *n*, %	807 (78.7%)	610 (75.9%)	1339 (78.1%)	133 (73.9%)	287 (76.1%)	.03
Partner taking care (hw) *M*, *SD*	16.54 (14.85)	15.82 (13.5)	18.84 (15.36)	13.9 (11.94)	14.59 (13.57)	.01***
Partner taking care (mth) *M*, *SD*	1.3 (1.4)	1.34 (1.42)	1.18 (1.35)	1.73 (1.76)	1.43 (1.55)	.01**
Family taking care (hw) *M*, *SD*	8.7 (9.6)	10.53 (12.46)	9.45 (11.18)	11.39 (9.72)	12.9 (16.56)	.01**
Family taking care (month) *M*, *SD*	2.01 (1.69)	1.75 (1.52)	1.81 (1.63)	1.7 (1.76)	1.91 (1.59)	<.01
Non-family taking care (hw) *M*, *SD*	5.9 (7.77)	6.07 (8.53)	4.5 (4.35)	6.14 (8.51)	5.06 (5.17)	0.01
Non-family taking care (mth) *M*, *SD*	3.04 (1.97)	2.62 (1.72)	2.81 (1.92)	3.57 (2.51)	3.44 (2.09)	0.02
Emotional life						
Love of the baby *M*, *SD*	1.38 (0.62)	1.36 (0.6)	1.26 (0.52)	1.33 (0.62)	1.45 (0.74)	.01***
Parental history						
Parental care (m.) *M*, *SD*	38.71 (6.54)	39.02 (6.26)	39.96 (5.86)	37.36 (7.22)	38.19 (7.22)	.01***
Parental care (f.) *M*, *SD*	38.97 (5.67)	38.19 (5.74)	39.06 (5.59)	37.31 (6.61)	38.45 (6.23)	.01***
Overprotection (m.) *M*, *SD*	22.9 (4.79)	22.25 (4.65)	21.69 (4.43)	23.52 (4.72)	23.54 (4.95)	.02***
Overprotection (f.) *M*, *SD*	22.51 (4.59)	23.77 (4.63)	22.23 (4.8)	24.71 (4.72)	23.16 (4.81)	.02***
Home stability (m.) *M*, *SD*	2.88 (0.53)	2.91 (0.52)	2.92 (0.54)	2.93 (0.51)	2.90 (0.55)	<.01
Home stability (f.) *M*, *SD*	2.93 (0.58)	2.90 (0.56)	2.92 (0.58)	2.91 (0.6)	2.93 (0.56)	<.01
Sexual abuse (m.) *M*, *SD*	0.38 (0.64)	0.41 (0.66)	0.29 (0.58)	0.41 (0.68)	0.47 (0.68)	.01***
Sexual abuse (f.) *M*, *SD*	0.11 (0.39)	0.17 (0.48)	0.1 (0.37)	0.24 (0.58)	0.16 (0.47)	.01***
Obstetric history						
N of previous pregnancies *M*, *SD*	1.79 (1.1)	1.84 (1.19)	1.73 (1.07)	2.09 (1.48)	2.06 (1.43)	.01***

N of children *M*, *SD*	1.05 (0.7)	1.02 (0.73)	0.99 (0.71)	1.21 (1.06)	1.12 (0.8)	.01*
N of miscarriage *M*, *SD*	1.21 (0.47)	1.16 (0.46)	1.19 (0.51)	1.16 (0.37)	1.17 (0.47)	<.01
N of abortion *M*, *SD*	1.31 (0.62)	1.38 (0.7)	1.26 (0.59)	1.27 (0.49)	1.38 (0.66)	.01
Health						
Alcohol use (m.) *M*, *SD*	0.18 (0.71)	0.16 (0.65)	0.13 (0.61)	0.14 (0.59)	0.2 (0.71)	<.01
Alcohol use (f.) *M*, *SD*	1.6 (1.3)	1.71 (1.36)	1.54 (1.34)	1.59 (1.33)	1.69 (1.39)	<.01*

Note. m = mother. f = father. hw = hours per week. mth = age of child in months.

* $p < .05$. ** $p < .01$. *** $p < .001$.

Table S11. Brown-Forsythe analysis of variance tests comparing the five classes along the offspring’s temperament (*M*, *SD*) that had weak associations

	Class 1: Mother has elevated depression, father is non-depressed	Class 2: Both mother and father have elevated depression	Class 3: Both mother and father are constantly non-depressed	Class 4: Both mother and father are constantly depressed	Class 5: Mother is constantly depressed, father has elevated depression	η^2
Calm	3.12 (0.77)	3.16 (0.79)	3.34 (0.73)	3.21 (0.72)	3.12 (0.82)	.02***
Chatty	2.82 (0.91)	2.82 (0.9)	2.86 (0.95)	2.96 (0.85)	2.9 (0.9)	<.01
Whiny	1.91 (0.77)	1.81 (0.76)	1.69 (0.71)	1.88 (0.75)	1.94 (0.78)	.02***
Demanding	2.53 (0.96)	2.46 (0.94)	2.28 (0.97)	2.53 (0.99)	2.55 (0.97)	.01***
Angry	1.62 (0.76)	1.53 (0.71)	1.47 (0.66)	1.7 (0.84)	1.66 (0.81)	.01***
Clingy	3.32 (0.66)	3.33 (0.68)	3.34 (0.66)	3.3 (0.71)	3.28 (0.71)	<.01
Lively	3.53 (0.6)	3.56 (0.57)	3.54 (0.62)	3.51 (0.61)	3.44 (0.69)	<.01*
Social	3.19 (0.82)	3.18 (0.83)	3.16 (0.85)	3.16 (0.86)	3.1 (0.91)	<.01
Closed	1.41 (0.58)	1.4 (0.55)	1.31 (0.52)	1.51 (0.66)	1.48 (0.64)	.01***
Stubborn	1.66 (0.78)	1.63 (0.78)	1.52 (0.73)	1.69 (0.82)	1.75 (0.86)	.01***
No interest	1.36 (0.61)	1.3 (0.57)	1.28 (0.57)	1.31 (0.55)	1.36 (0.61)	<.01**
Satisfied	3.35 (0.61)	3.41 (0.6)	3.51 (0.59)	3.35 (0.69)	3.3 (0.69)	.02***
Bright	3.4 (0.63)	3.38 (0.66)	3.43 (0.62)	3.41 (0.66)	3.33 (0.73)	<.01

* $p < .05$. ** $p < .01$. *** $p < .001$.

Table S12. Brown-Forsythe analysis of variance tests comparing the five classes along the offspring's mental health (*M*, *SD*) that had weak associations

	Class 1: Mother has elevated depression, father is non-depressed	Class 2: Both mother and father have elevated depression	Class 3: Both mother and father are constantly non-depressed	Class 4: Both mother and father are constantly depressed	Class 5: Mother is constantly depressed, father has elevated depression	η^2
Stressful life events (15y)	0.43 (0.33)	0.45 (0.33)	0.38 (0.31)	0.51 (0.41)	0.55 (0.47)	.02***
Stressful life events (18y)	0.47 (0.27)	0.48 (0.28)	0.42 (0.28)	0.52 (0.33)	0.54 (0.29)	.02*
SDQ: internalizing[1] (11y)	8.27 (4.1)	8.15 (4.04)	7.33 (3.96)	8.84 (4.32)	9.28 (4.14)	.02***
SDQ: externalizing[1] (11y)	9.83 (4.48)	9.51 (4.5)	8.81 (4.59)	10.67 (4.73)	10.37 (4.82)	.02***
SDQ: internalizing[2] (15y)	5.39 (3.09)	5.67 (3.34)	4.8 (3.28)	6.05 (3.14)	6.11 (3.41)	.02***
SDQ: externalizing[2] (15y)	6.66 (3.3)	6.81 (3.41)	6.18 (3.32)	6.78 (3.45)	7.59 (3.96)	.01***
SDQ: internalizing[2] (18y)	5.44 (3.04)	5.89 (3.28)	5.21 (3.21)	6.08 (2.93)	6.48 (3.46)	.02
SDQ: externalizing[2] (18y)	6.5 (3.43)	5.95 (3.05)	5.9 (3.37)	5.95 (2.86)	7.15 (3.74)	.01
Self-esteem (15y)	29.69 (4.65)	28.85 (4.39)	29.89 (4.38)	28.54 (4.2)	28.34 (4.59)	.02***
Self-esteem (18y)	30.37 (5.26)	30.34 (4.71)	30.62 (4.87)	29.67 (4.85)	30.52 (4.09)	<.01
Self-esteem (19y)	30.48 (5.69)	30.8 (5.39)	31.68 (5.92)	29.63 (4.19)	32.91 (3.25)	.02
Satisfaction with life (15y)	17.47 (4.19)	17.38 (3.73)	18 (3.82)	16.97 (3.97)	16.55 (4.21)	.01**
Satisfaction with life (19y)	17.61 (4.78)	17.97 (4.45)	18.06 (4.48)	17.02 (3.63)	18.56 (3.9)	.01

Note. M = mean. *SD* = standard deviation. y = years of age. SDQ = Strength and Difficulties Questionnaire.

[1] SDQ items were measured on a 0 to 3 scale.

[2] SDQ items were measured on a 0 to 2 scale.

* $p < .05$. ** $p < .01$. *** $p < .001$.

a

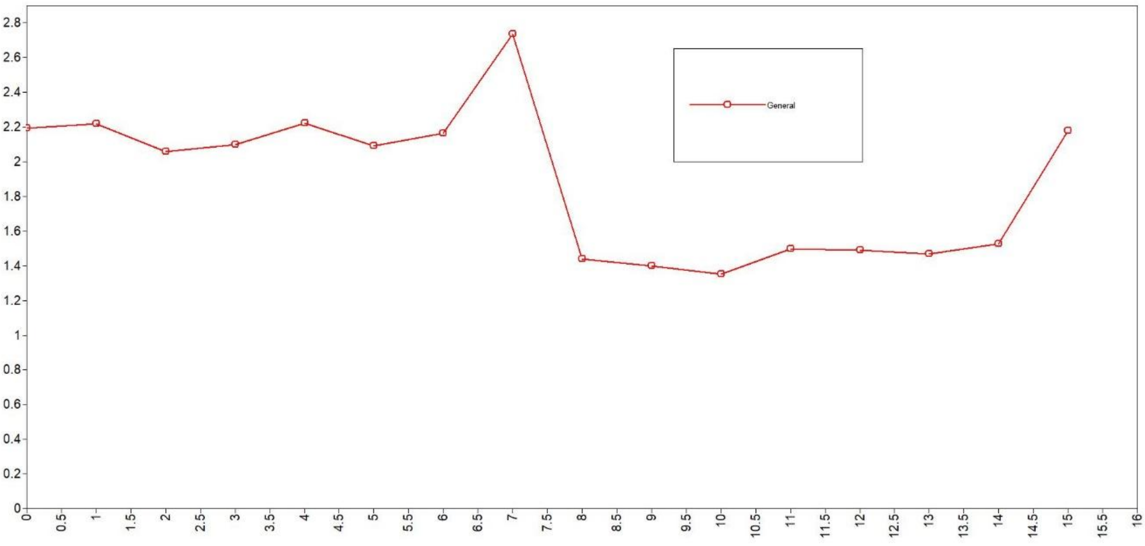
General

b

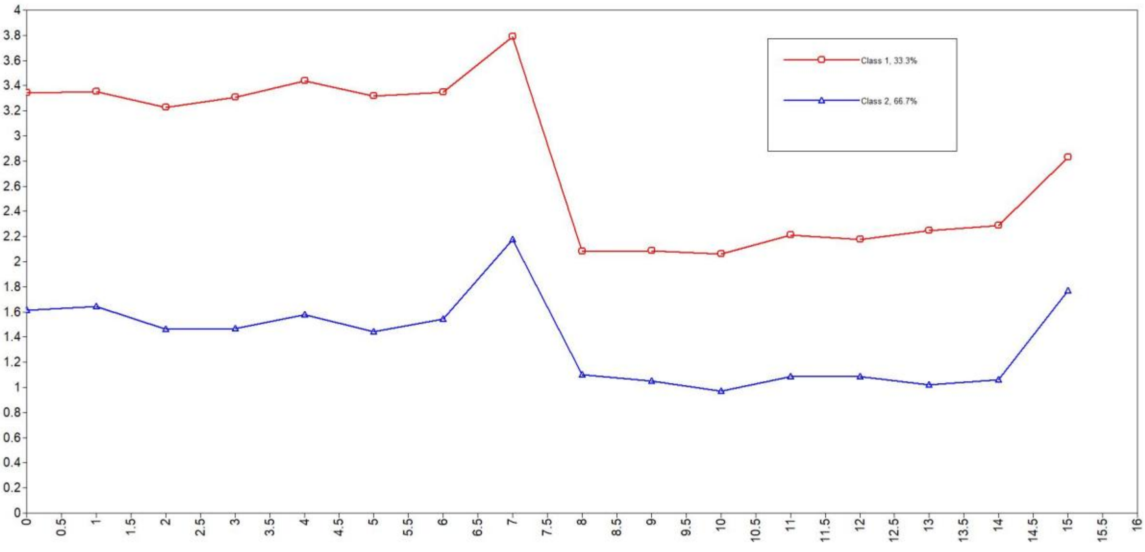
Class 1, 33.3%
Class 2, 66.7%

c

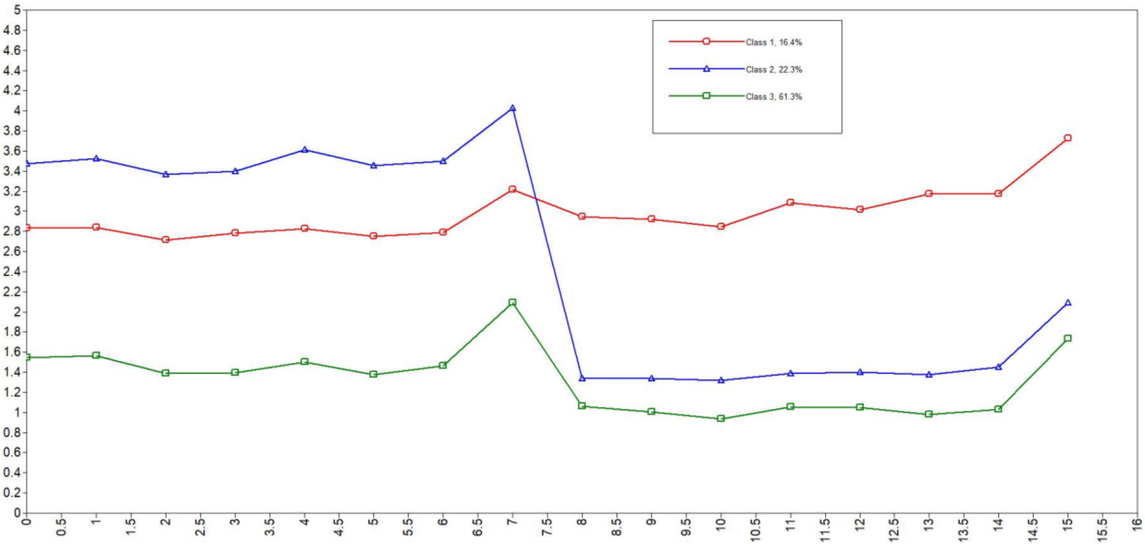
Class 1, 16.4%
Class 2, 22.3%
Class 3, 61.3%

d

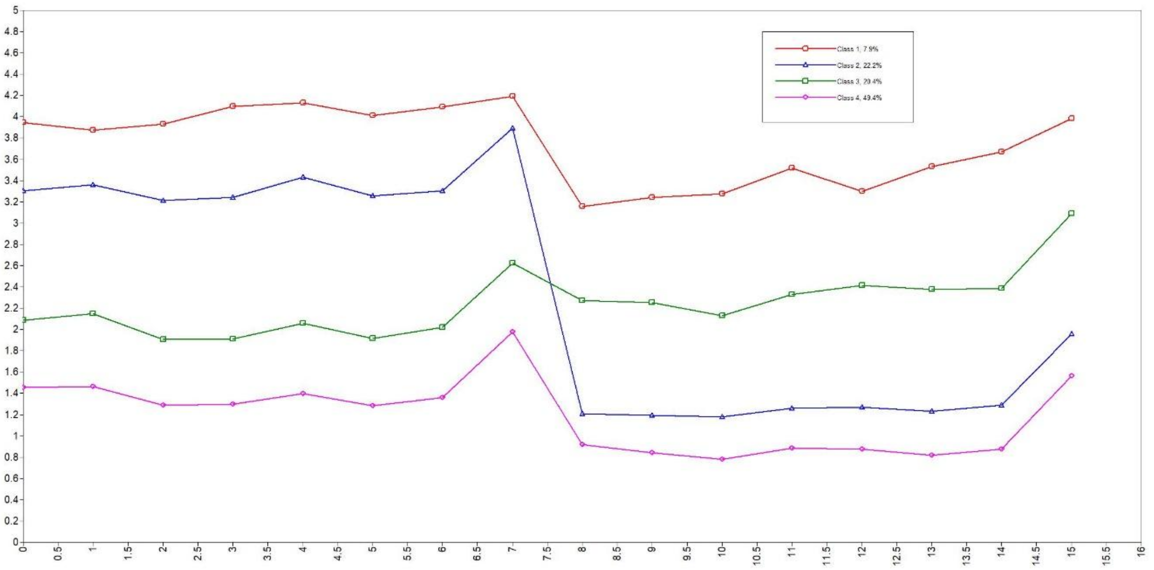
Class 1, 7.9%
Class 2, 22.2%
Class 3, 20.4%
Class 4, 49.4%

e

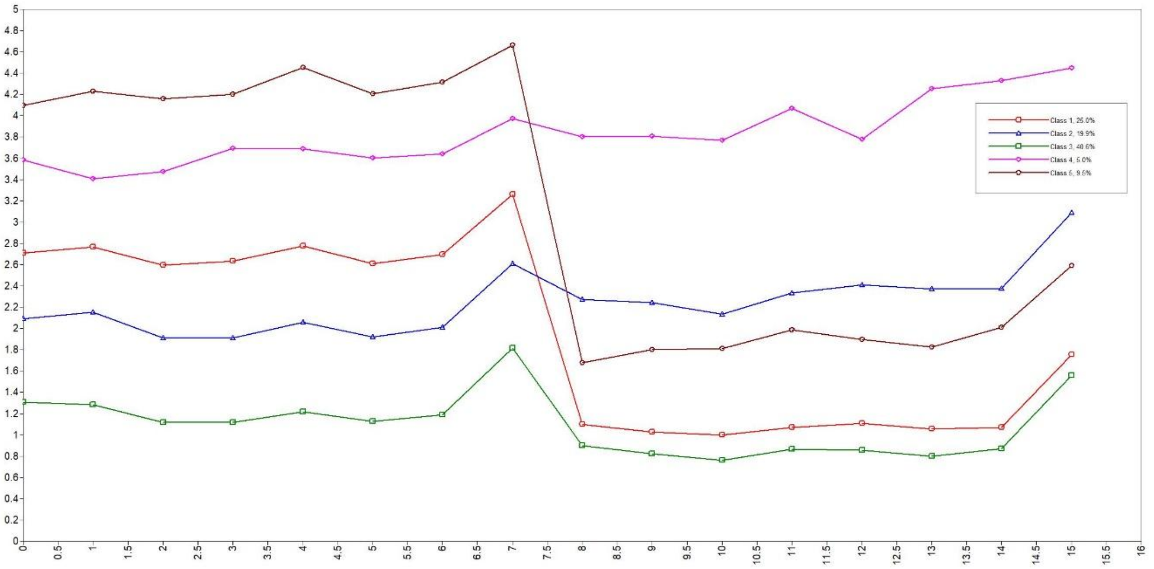
Class 1, 25.0%
Class 2, 19.9%
Class 3, 40.6%
Class 4, 5.0%
Class 5, 9.5%

f

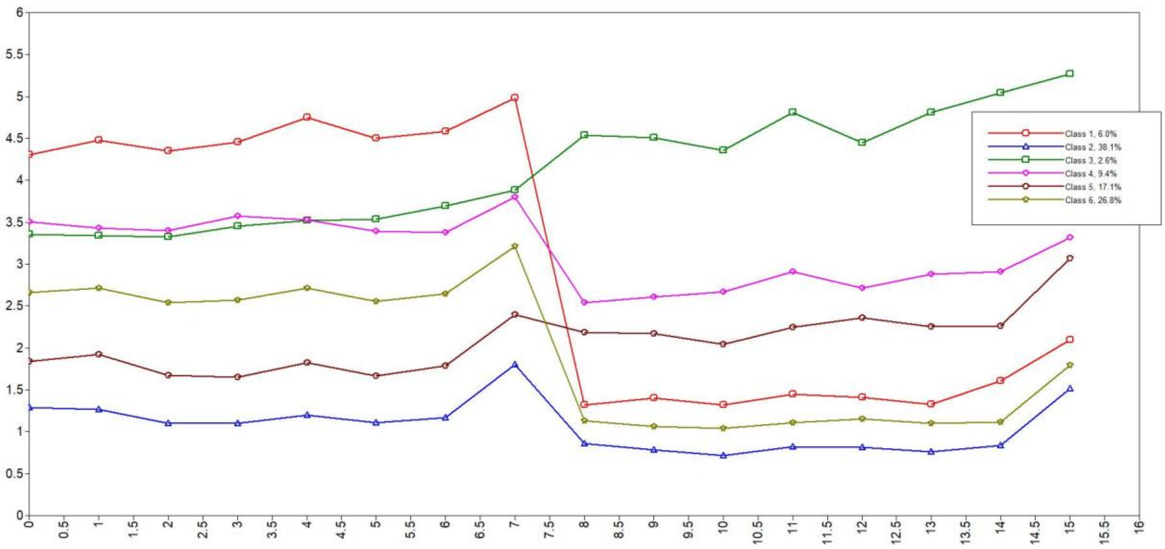

Figure S1. Mean depressive symptoms of mothers and fathers (left: 0, 1, 2, 3, 4, 5, 6, and 7 points on the x axis; and right: 8, 9, 10, 11, 12, 13, 14 and 15 points on the x axis within each diagram, respectively) in each model extracting 1 to 6 classes. **a**: latent growth model on the overall sample; **b**: 2-class model; **c**: 3-class model; **d**: 4-class model; **e**: 5-class model; **f**: 6-class model.

a

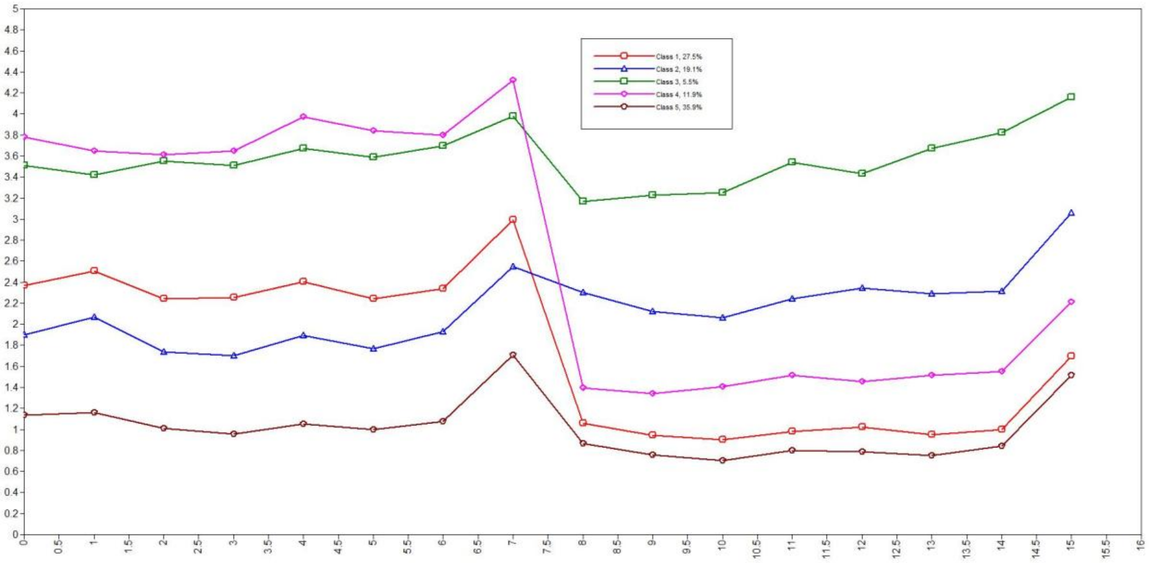

b

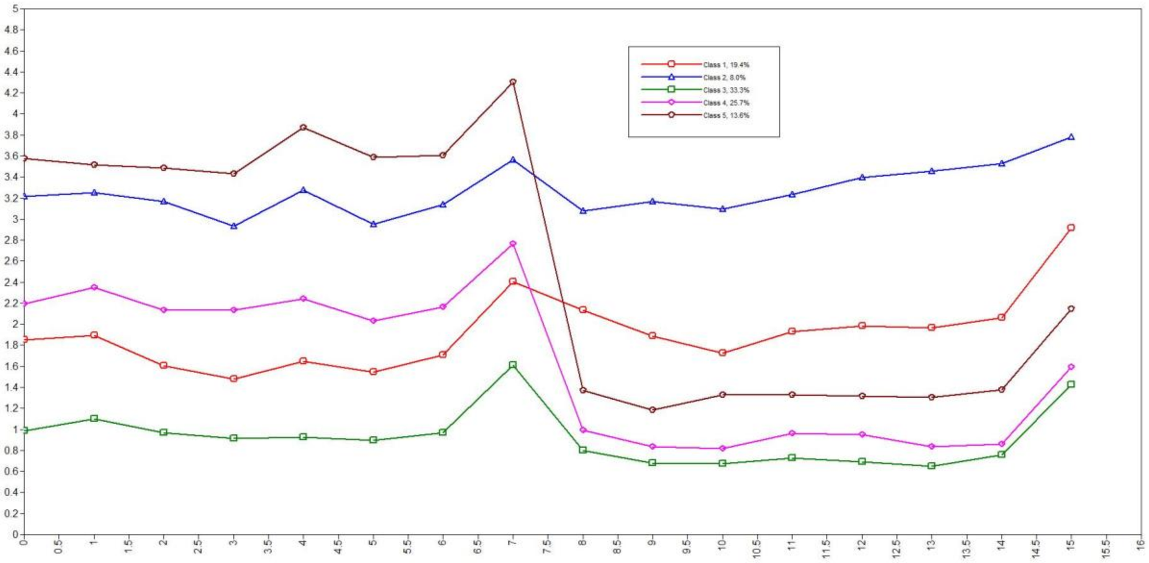

Figure S2. Patterns yielded from the sensitivity analysis obtained on subsets of the sample with varying data coverage. Mean depressive symptoms of mothers and fathers (left: 0, 1, 2, 3, 4, 5, 6, and 7 points on the x axis; and right: 8, 9, 10, 11, 12, 13, 14 and 15 points on the x axis within each diagram, respectively) in each model extracting 5 classes. **a**: good data coverage (both mother and father had at least 6 time-points out of 8, *n* = 2528); **b**: full data coverage (both mother and father had all 8 data points covered, *n* = 951).

References

Akaike, H. (1973). Information theory as an extension of the maximum likelihood principle. In B. N. Petrov & F. Csaki (Eds.), *Second international symposium on information theory* (p. 267). Budapest, Hungary: Akademiai Kiado.

Brown, T. A. (2006). *Confirmatory factory analysis for applied research.* Guilford Press.

Lo, Y., Mendell, N., & Rubin, D. (2001). Testing the number of components in a normal mixture. *Biometrika*, *88*, 767–778.

McLachlan, G., & Peel, D. (2004). *Finite mixture models*. New York, NY: Wiley.

McNeish, D., & Harring, J. (2020). Covariance pattern mixture models: Eliminating random effects to improve convergence and performance. *Behavior Research Methods, 52*, 947-979.

McNeish, D., Harring, J. R., & Bauer, D. J. (2023). Nonconvergence, covariance constraints, and class enumeration in growth mixture models. *Psychological Methods, 28*, 962-992.

Schwarz, G. (1978). Estimating the dimension of a model. *The Annals of Statistics*, *6*, 461–464.

Van De Schoot, R., Sijbrandij, M., Winter, S. D., Depaoli, S., & Vermunt, J. K. (2017). The GRoLTS-checklist: guidelines for reporting on latent trajectory studies. *Structural Equation Modeling: A Multidisciplinary Journal*, *24*, 451-467.

Vuong, Q. (1989). Likelihood ratio tests for model selection and non-nested hypotheses. *Econometrica, 57*, 307-333.
